# Rice matrix metalloproteinase OsMMP1 plays pleiotropic roles in plant development and symplastic-apoplastic transport by modulating cellulose and callose depositions

**DOI:** 10.1038/s41598-018-20070-4

**Published:** 2018-02-09

**Authors:** Prabir Kumar Das, Rupam Biswas, Nazma Anjum, Amit Kumar Das, Mrinal K. Maiti

**Affiliations:** Department of Biotechnology, Indian Institute of Technology Kharagpur, Kharagpur, 721302 India

## Abstract

Matrix metalloproteinases (MMPs) are well-known proteolytic enzymes in animal systems and play roles in tissue differentiation, growth, and defence. Although a few plant MMPs have been reported, their exact functions in development and growth remain elusive. In this study, we characterized the promoter and coding sequence of OsMMP1, one of the putative MMP genes in rice (*Oryza sativa*). The OsMMP1 catalytic domain is structurally similar to human MMPs with respect to cofactor orientation as predicted by homology modeling. Bacterially expressed recombinant OsMMP1 showed protease activity with bovine serum albumin and gelatin as substrates. Analyses of transcript accumulation and promoter-reporter gene expression revealed that OsMMP1 is spatio-temporally expressed in vegetative and reproductive parts of plants. The plasma membrane-localized OsMMP1 protease affected plant development upon heterologous expression in tobacco and endogenous gene silencing in rice. Transgenic tobacco plants expressing OsMMP1 showed enhanced deposition of cellulose and callose, leading to impairment of symplastic and apoplastic translocations. Moreover, transgenic tobacco tissues exhibited tolerance to oxidative stress-inducing agent by confining the area of tissue death owing to callose lining. Collectively, these findings demonstrate the involvement of a plant MMP in growth, organ differentiation, and development in relation to cell wall modification.

## Introduction

Plant organogenesis and morphogenesis are highly coordinated activities occurring around the cell wall, leading to visible development and growth. Development is the result of complex gene-gene interactions, waves of hormonal signaling, sensing and responding towards various environmental stimuli in different combinations^1^. Extracellular matrix (ECM), a cell surface continuum beyond the cell wall, play a vital role in cell adhesion, cell-cell communication, cell wall modification, and protection against stresses. Interactions among neighbouring cells through transport of water, nutrients, phytohormones, and signaling molecules are essential for morphogenesis, development, and growth. Maintenance of cell shape and structural integrity of the cell wall and ECM components are critical in these processes. In recent years, significant works have been carried out on maintenance mechanism of plant cell wall integrity^2^; still, a big void exists in comprehensive understanding. Cell wall and ECM are structurally and functionally dynamic in nature, and their constituents play important roles in maintaining cell wall integrity. Various proteases of cell wall help in releasing signaling peptides and control the degradation or maturation of cell wall modifying enzymes: hence, they play critical roles in modulating cell wall constituents during growth and development^3^. On the other hand, the matrix metalloproteinase (MMP) is a very important protease of ECM, and the exact roles of plant MMPs are not very clear.

MMPs are well characterized in animal systems as proteolytic enzymes of metzincin superfamily^4^ and play crucial roles in regulating the ECM composition. Multiple isoforms of these proteases are present in a given species, and they share certain common structural and functional domains within and across the species. MMPs have roles in normal and stress-induced physiological processes, including embryogenesis, wound healing, inflammation, arthritis, and cancer^5–9^. MMPs are synthesized as pre-proproteins and secreted in latent forms, which require activation before they become functional. The enzymatically active MMP is a zinc-dependent endopeptidase characterized by the signature motif **H**EXG**H**XXGXX**H** in the catalytic domain, wherein three histidine residues coordinate the Zn^2+^ in the active site of the enzyme. These proteins have a conserved methionine turn as well as a conserved cysteine switch sequence PR**C**GXPD, where the cysteine of this domain interacts with the catalytic Zn^2+^ ^10,11^. The Cys residue ligates the catalytic zinc, thus maintains the latency of the inactive pro-enzymes^12^.

The first MMP activity in plant systems was reported in soybean leaves and termed as Azocollase, an EDTA-sensitive Azocoll-degrading enzyme^13^, which was later characterized as metalloendoproteinase (SMEP1)^14^. Since then, only a few plant MMPs have been studied to reveal their proteolytic property and expression profile in Arabidopsis^15,16^, cucumber^17^, *Medicago truncatula*^18^, soybean^19^, and tobacco^20^. Gene expression analyses suggested that these proteases are critical for ECM remodeling and environmental stresses.

Since the exact roles of plant MMPs in developmental physiology remain elusive and this class of protease has potential biotechnological application in the crop plant, it is pertinent to comprehend its detailed functions. We identified a few hypothetical MMP-like proteins in *indica* rice (*Oryza sativa*) through bioinformatics using the reported tobacco (*Nicotiana tabacum*) NtMMP1 protein (GenBank: ABF58910)^20^ as a query. Among three significant hits (Supplementary Figs S1–S3), the putative rice MMP exhibiting the highest homology with NtMMP1 was designated as OsMMP1. In the present study, we aimed to decipher the physiological function of rice matrix metalloproteinase OsMMP1. We asked the following questions: (1) Is the newly cloned gene product structurally homologous to other characterized MMPs? (2) In which tissues or organs is the *OsMMP1* gene expressed and in which sub-cellular organelle or compartment is the protein localized to impart its protease activity? (3) Does this protein have any role in the maintenance of cell wall integrity? (4) What phenotypic changes might occur upon heterologous expression (*gain-of-function*) and endogenous down-regulation (*loss-of-function*) of OsMMP1? From our findings and analyses, we document that the OsMMP1 protease has pleiotropic roles in plant’s growth, development, and adaptation to environmental stress.

## Results

### OsMMP1 protein has similarity with other MMPs in terms of structural features and proteinase activity

**Figure 1:**
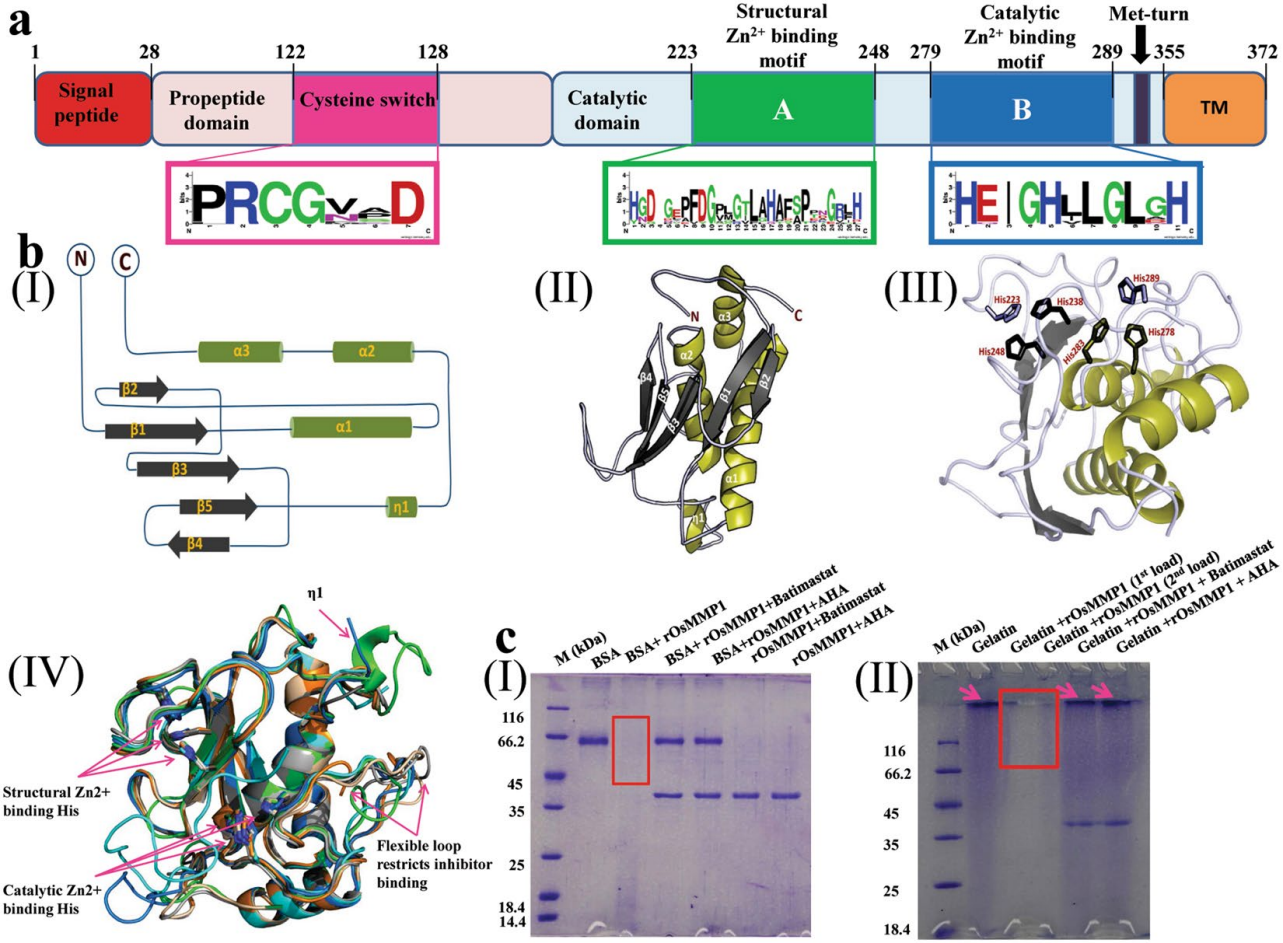
Structure prediction and proteolytic activity assay of OsMMP1. (**a**) Schematic diagram of the predicted domains of OsMMP1 protein and WebLogo plot of the consensus sequence of cysteine switch, structural and catalytic Zn^2+^-binding motifs. The consensus sequence was determined based on the frequency of each amino acid in corresponding position of the amino acid sequence of the aligned MMPs using WebLogo design tool (http://weblogo.berkeley.edu/logo.cgi). WebLogo plot reveals that the niches having the cysteine switch (PRCGVAD) and catalytic Zn^2+^-binding motif (HEIGHLLGLGH) are highly conserved in comparison with the structural Zn^2+^-binding motif (HGDGEAFDGPLGTLAHAFSPTDGRFH). The diagram is not drawn to the scale. The number indicates the position of amino acids spanning the critical domains and motifs. (**bI**) Topology diagram of the OsMMP1 catalytic domain displays four parallel β-sheets, one anti-parallel β sheet, three α- helices and a 3_10_-helix (η1). (**bII**) Cartoon representation of the model structure of OsMMP1 catalytic domain. (**bIII**) The 3D orientation of six His residues participating in the coordination bond with two Zn^2+^ ions. (**bIV**) Structural superimposition of OsMMP1 (green) with human MMP1 (red), MMP2 (marine), MMP3 (wheat), MMP9 (cyan), MMP10 (orange), and MMP13 (grey) shows the conserved folds and the conserved secondary structures. (**cI**,**II**) Analysis of the products formed after protease activity of the recombinant OsMMP1 (rOsMMP1). Rectangular boxes indicate the proteolytic degradation of (**cI**) BSA and (**cII**) gelatin. The arrow indicates the (**cII**) gelatin protein band. Lane M: protein molecular weight marker. The degradation of (**cI**) BSA is prominent in the 3^rd^ lane, but the rOsMMP1 band is absent due to its autocatalytic property. Similarly, the degradation of (**cII**) gelatin is prominent in the 3^rd^ and 4^th^ lanes, but the rOsMMP1 band is absent due to its autocatalytic property. The MMP inhibitors, Batimastat and acetohydroxamic acid (AHA) are efficient in inhibiting the proteolytic and autocatalytic activities of rOsMMP1. Effects of both the inhibitors are quite similar as both of them completely inhibit the activity of rOsMMP1 but the concentration of AHA is 25 times higher than Batimastat. Full-length gels of **cI** and **cII** are presented in Supplementary Figs S16, and S17, respectively.

The full-length coding DNA sequence (CDS) of *OsMMP1* was PCR-amplified from the *indica* rice cultivar IR64, cloned and sequenced. Sequence alignment of the 372-amino acid OsMMP1 with *Arabidopsis thaliana* At1-MMP, *Glycine max* SMEP1, *Cucumis sativus* Cs1-MMP2, *Solanum lycopersicum* SL2-MMP and SL3-MMP, and *Nicotiana tabacum* NtMMP1 revealed sequence similarity in the four common domains (Supplementary Fig. S4). These four crucial domains are the N-terminal signal peptide, a propeptide domain, a catalytic domain, and a C-terminal transmembrane (TM) domain (Fig. 1a). *In-silico* study of OsMMP1 disclosed the presence of a signal peptide of first 28 amino acids at the N-terminus with a cleavage site between Ala^28^ and Phe^29^, and a conserved methionine known as Met-turn just before the C-terminal TM domain. Moreover, the propeptide domain contains a cysteine-switch motif, and the catalytic domain consists of two Zn^2+^-binding motifs (Fig. 1a).

Since no crystal structure of plant MMP is available, we attempted to model OsMMP1 using existing crystal structures of human MMPs in the RCSB-PDB database (http://www.rcsb.org/pdb/home/home.do) as a template. The homology model of the OsMMP1 catalytic domain consists of three α-helices and a twisted five-stranded β-sheet in a β1-α1-β2-β3-β4-β5-η1-α2-α3 topology (Fig. 1bI,bII). The catalytic domain of OsMMP1 contains two conserved Zn^2+^-binding motifs, each having three characteristic His residues (Fig. 1bIII). The 3D model of OsMMP1 was superimposed on the crystal structure of human MMP1 (PDB id: 1SU3), MMP2 (PDB id: 1EAK), MMP3 (PDB id: 1G49), MMP9 (PDB id: 1L6J), MMP10 (PDB id: 1Q3A), and MMP13 (PDB id: 4G0D). It was found that the model structure of OsMMP1 is highly homologous to human MMPs, though the sequence identity is below 50%. The functionality of a class of enzyme is determined by the topology of the catalytic domain including the spatial arrangement of the conserved active site residues. Structural superimposition (Fig. 1bIV) revealed that OsMMP1 has a comparable root mean square deviation value (0.48 Å for 159 C^α^ atoms among 175 aligned C^α^ atoms) with MMP10, in spite of lowest sequence identity (38%) among six human MMPs used for the study (Supplementary Table S2). This signifies that the topology of the catalytic domain is highly conserved among the MMP superfamily. The main structural difference in OsMMP1 is within the loop region (from Ala^251^ to Asp^272^) connecting β5 and α2. This flexible part of OsMMP1 has a short 3_10_-helix (η1), which is absent in the superimposed human MMPs. Another structural difference in OsMMP1 lies in the flexible loop region from Arg^302^ to Lys^308^ that does not superimpose on the corresponding loop structure of human MMPs. Previously, from the crystal structures of human MMP3 and MMP10, it was predicted that this intrinsically flexible loop may be the key reason to restrict the success in designing MMP-specific inhibitors^21^.

The near full-length CDS of *OsMMP1* covering 29^th^ to 323^rd^ amino acids was expressed in *Escherichia coli* using the T7 polymerase-promoter system. Although the protein was expressed in the insoluble form, the refolding experiment was performed to bring the recombinant His-tagged OsMMP1 (rOsMMP1) in soluble form; and the purified protein was verified by western blotting using anti-OsMMP1 antibody (Supplementary Fig. S5). To check the enzymatic activity, the refolded rOsMMP1 was incubated overnight with either BSA or gelatin followed by SDS-PAGE. The degradation of BSA (Fig. 1cI) and gelatin (Fig. 1cII) was clearly visible on the gel, confirming the protease activity of the newly cloned *OsMMP1* gene product.

### *OsMMP1* gene is expressed constitutively during plant growth and development

**Figure 2:**
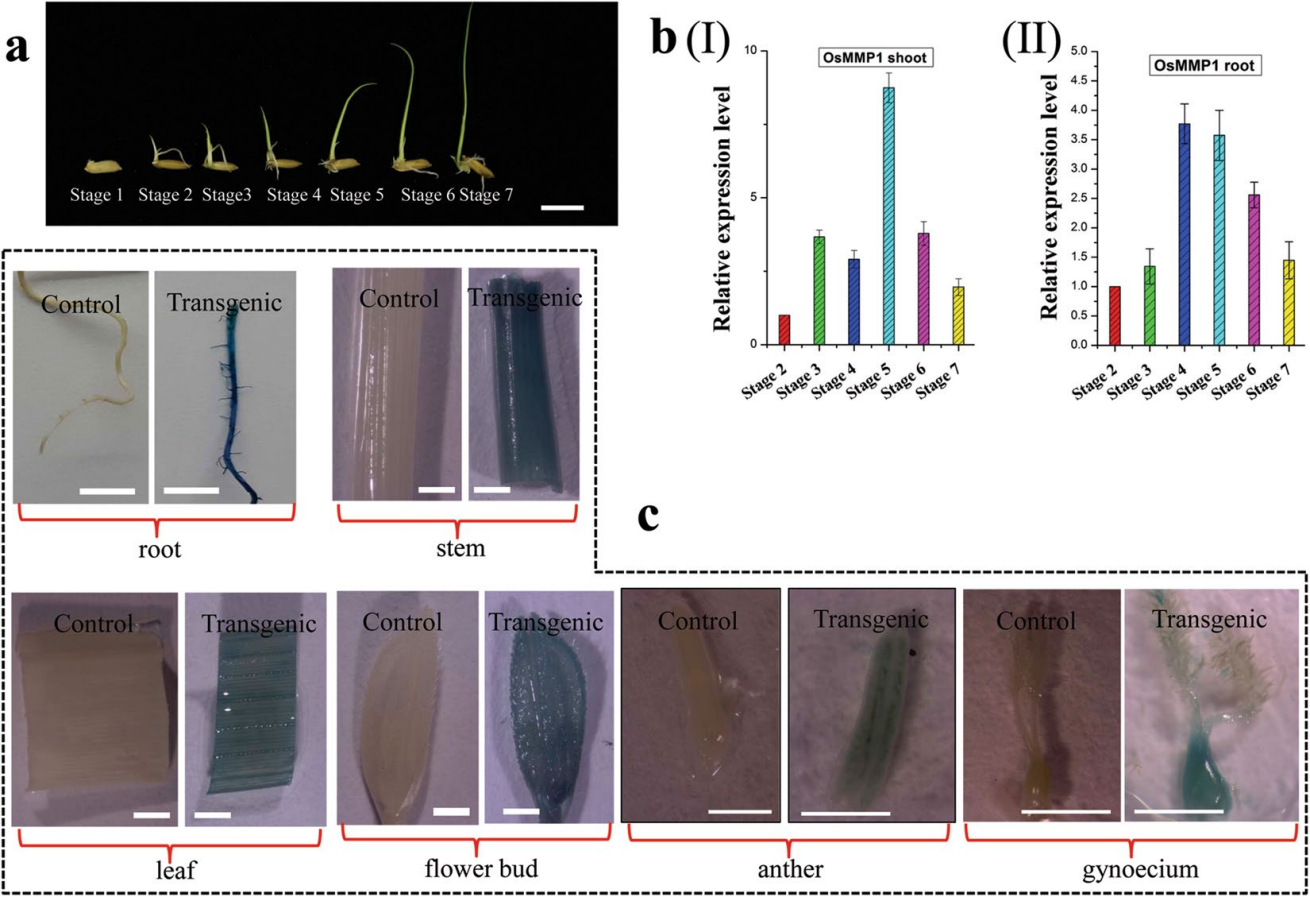
Spatio-temporal expression profile of *OsMMP1* gene. (**a**) Photograph shows developmental stages of rice seedlings harvested for RNA isolation. Scale bars = 10 mm. (**bI**,**II**) Stage-wise relative quantification of the *OsMMP1* gene expression in rice shoot and root tissues by qRT-PCR. Gene expression data were normalized against the internal control rice actin1 gene and calculated relative to the stage 2 expression value that was taken as one. (**c**) Micrographs of the *OsMMP1* promoter-driven *GUS* reporter expression in transgenic rice lines show GUS activity in root, stem, leaf, flower bud, anther, and gynoecium. All the lines of OsMMP1promoter:GUS transgenic rice were tested for GUS reporter expression, and were found to be consistently GUS positive in the tissues examined. They were also tested in different generations (T0 − T3). Here, all the GUS positive panels are from one transgenic line of T3 generation for proper representation. Scale bar for root = 50 mm, stem = 10 mm, leaf = 10 mm, flower bud = 10 mm, anther = 1 mm, and gynoecium = 1 mm. In all bar graphs, data are expressed as mean ± SD (n = 3).

To recognize the possible association of the OsMMP1 protease with plant development, two different experimental strategies were employed. Firstly, the transcript accumulation pattern of OsMMP1 was monitored during the development of rice plant by qRT-PCR. Six early developmental stages of rice seedling were selected (Fig. 2a), and RNA samples were isolated from root and shoot tissues of each stage. We found that the *OsMMP1* gene expression varies among the developmental stages (day-to-day) for both tissues (Fig. 2bI,bII), implying that the expression is developmental stage-dependent in native host rice.

Next, to understand the spatio-temporal expression pattern of the *OsMMP1* gene, the promoter segment was studied through *in-silico* analysis (Supplementary Fig. S6) and *in-planta* expression of the reporter gene. To examine the tissue-specific expression of OsMMP1 in the native host, transgenic rice lines were generated with the *Agrobacterium* binary plasmid containing the β-glucuronidase (*gusA/uidA/GUS*) reporter gene under the control of 2 kb putative promoter of *OsMMP1* (Supplementary Fig. S7). Since the *GUS* gene was directly under the control of promoter without any 5′ part of the *OsMMP1* CDS, it was assumed that the expression of GUS reporter would only specify the tissue specificity but not the sub-cellular localization of the protein. Reporter gene expression revealed a strong GUS signal in root, stem, leaf, flower bud, anther, and gynoecium of transgenic rice lines (Fig. 2c), suggesting that *OsMMP1* is constitutively expressed in all tissue types of the rice plant. The same 2 kb *OsMMP1* promoter was fused with the green fluorescent protein (GFP) gene, and introduced in heterologous tobacco plant (Supplementary Fig. S8). The transgenic tobacco lines showed the expression of GFP in radicle of germinating seed, cross-sections of stem and stigma, and in transverse sections of anther (Supplementary Fig. S9). GUS and GFP expressions in both vegetative and reproductive parts of different transgenic plant species confirmed the probable association of OsMMP1 with the development and morphogenesis.

### Heterologous expression in tobacco revealed that the plasma membrane-localized OsMMP1 affects plant developmental process

To investigate the sub-cellular localization of OsMMP1 and *gain-of-function* phenotype due to OsMMP1 expression, transgenic tobacco lines were generated using the *Agrobacterium* binary plasmid containing the *OsMMP1* CDS driven by the enhanced 2XCaMV35S promoter (Supplementary Fig. S10).

**Figure 3:**
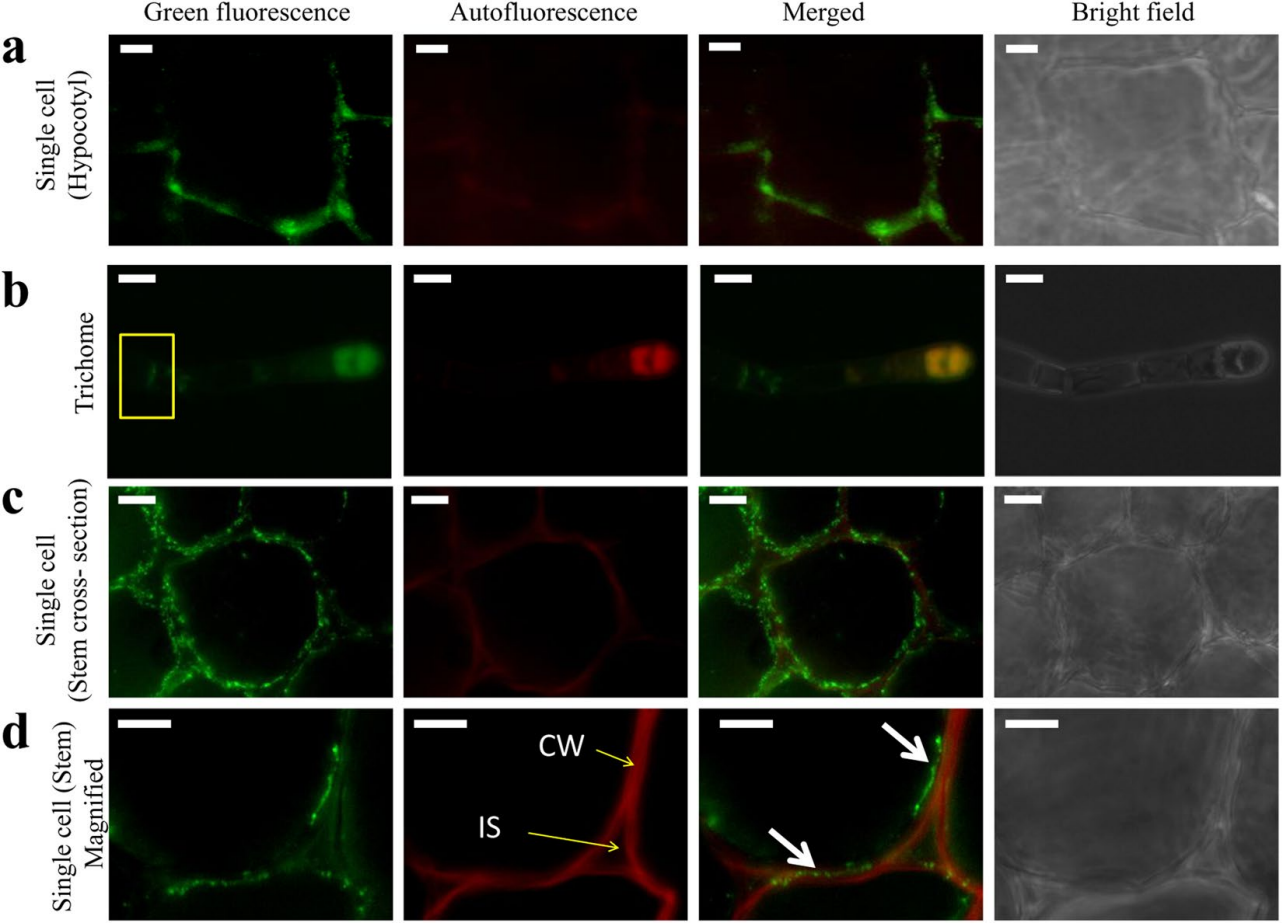
Immunolocalization of OsMMP1 in transgenic tobacco tissues. Fluorescence micrographs show green fluorescence from the cell wall adjacent area of (**a**) hypocotyls, (**b**) trichome (junction of two cells is indicated by box), and (**c**,**d**) stem cross-sections. A single cell from the stem cross-section clearly depicts the intense fluorescence is localized in the plasma membrane (indicated by arrow). Anti-OsMMP1 primary antibody was used to detect the heterologously expressed OsMMP1 in tobacco plant. Green fluorescence is due to the FITC-tagged secondary antibody attached to the anti-OsMMP1 primary antibody. CW, cell wall; IS, intercellular space. Scale bar = 10 µm.

Immunohistochemistry was carried out in transgenic tobacco tissues using western blot-detected positive samples (Supplementary Fig. S11) to examine the sub-cellular localization of OsMMP1. Analysis of transgenic lines revealed that the heterologous protein is localized in the plasma membrane of different cells (Fig. 3). However, no significant cross-reactivity of anti-OsMMP1 antibody was detected in non-transformed control (Supplementary Fig. S12). Immunolocalization of OsMMP1 was observed in different tissues, like hypocotyls (Fig. 3a), trichomes (Fig. 3b), and stem cross-sections (Fig. 3c,d). Interestingly, we noticed that the localization of OsMMP1 is exclusively at the junction of two cells in trichomes (Fig. 3b).

**Figure 4:**
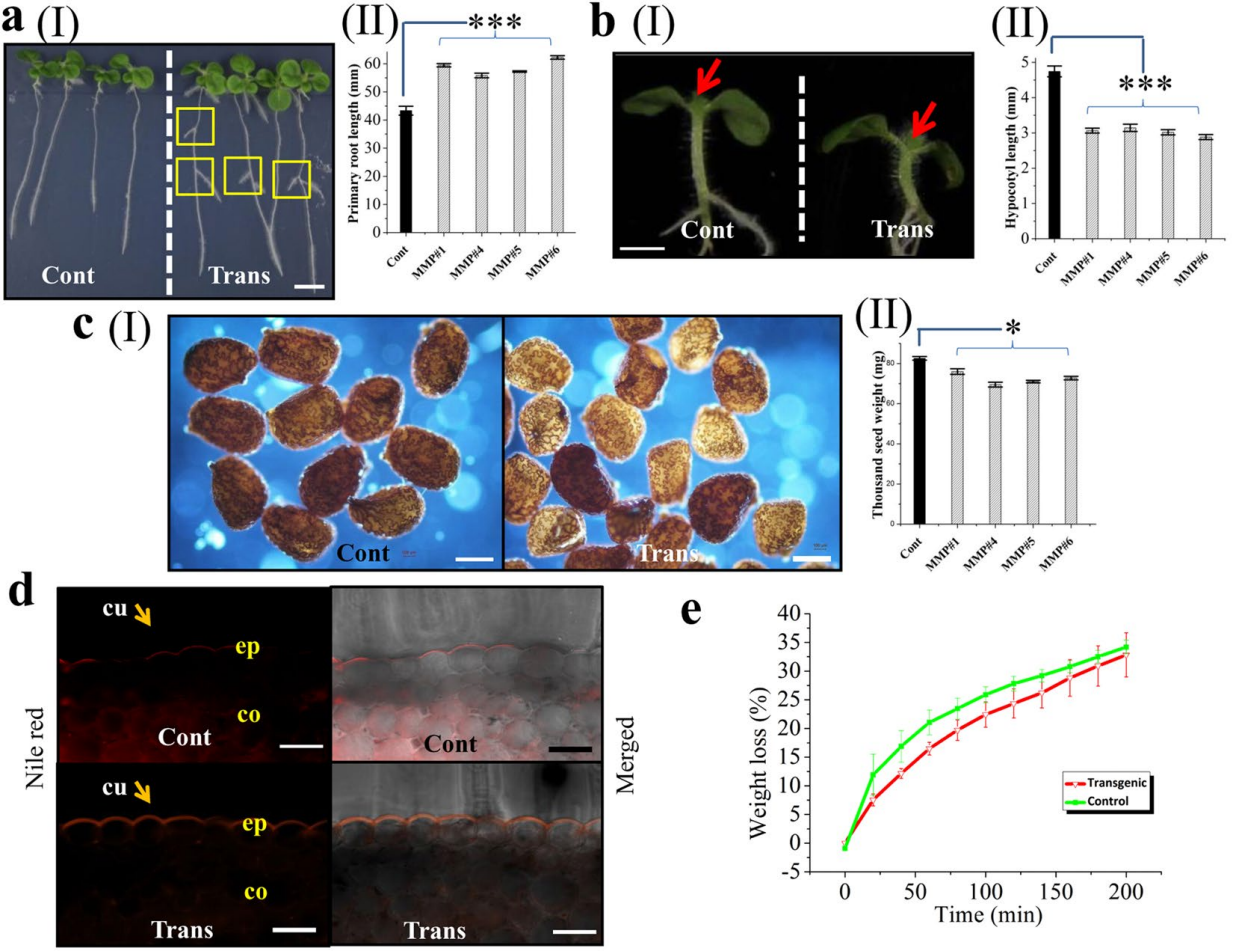
Heterologous expression of OsMMP1 affects developmental phenotypes in transgenic (Trans) tobacco compared with the non-transformed control (Cont) plants. (**aI**) Photograph of transgenic tobacco seedlings (each plantlet representing a single transgenic line) shows early lateral root emergence (rectangular boxes) and longer primary root length. Scale bar = 10 mm. (**aII**) Comparison of primary root length (in mm) in control and transgenic seedlings. (**bI**) Photograph exhibits shorter hypocotyl length in transgenic seedlings. Arrow mark denotes that the upper part from the first node was removed out for photography purpose. Scale bar = 2 mm. (**bII**) Comparison of hypocotyl length (in mm) in control and transgenic seedlings. (**cI**) Micrographs show smaller seed size in transgenic lines. Scale bar = 300 µm. Bright field micrographs were taken using Nikon ECLIPSE 50*i* upright microscope, and images were analysed with NLS-Elements D 3.2 software. (**cII**) Comparison of thousand seed weight (mg) in control and transgenic lines. (**d**) Micrographs show thicker cuticle layer at the epidermal cell surface (denoted by arrow) in transgenic stem cross-section. Stem sections were stained with Nile Red, and images were captured with an epifluorescence microscope. cu, cuticle; co, collenchymas; ep, epidermis. Scale bar = 50 µm. (**e**) Measurement of water loss indicates transgenic plants are relatively resistant to cuticular transpiration. The whole leaves of the dark-acclimated plants were excised and soaked in water for 60 min in dark^26^. Leaves were blotted and weighed at the indicated time points. In all bar graphs and line graph, data are expressed as mean ± SE of 10 observations (each transgenic line) for (**aII**) and (**bII**); as mean ± SE of 5 observations (each transgenic line) for (**cII**); as mean ± SE of 3 observations (each transgenic line) for (**e**). Data are subjected to Student’s *t*-test: ***P < 0.001 for (**aII**) and (**bII**); *P < 0.05 for (**cII**).

Analysis revealed different phenotypic changes in the stable transgenic tobacco lines expressing *OsMMP1*. Transgenic lines showed the early emergence of lateral roots with significantly longer primary root length (Fig. 4aI,aII). Interestingly, we observed that although the overall growth of the transgenic plant looks superior with respect to the control plant, the length of hypocotyls in transgenic lines was significantly shorter (Fig. 4bI,bII). Seed weight decreased significantly in transgenic tobacco (Fig. 4cI,cII). A thicker layer of cuticle was found on the aerial epidermal cell of the transgenic plant compared with the control (Fig. 4d). To evaluate whether the modified cuticle layer has any impact on the physiology, the loss of water was measured in transgenic and control leaves at different time intervals. The result showed that the transgenic leaf sample exhibited lower water loss rate compared to the control due to slower cuticular transpiration (Fig. 4e).

### RNAi-mediated *OsMMP1* gene silencing in autologous host rice causes phenotypic changes

**Figure 5:**
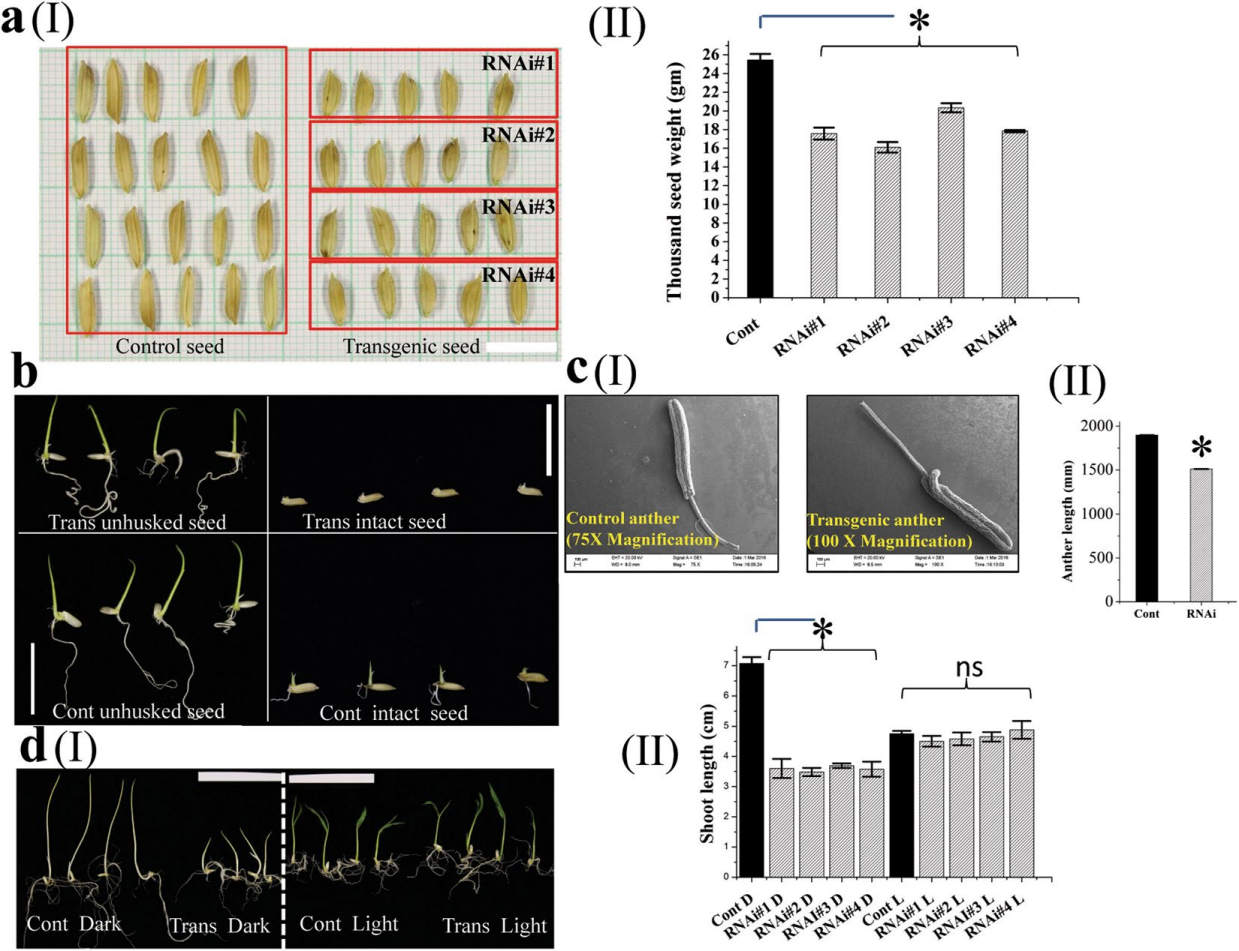
Endogenous *OsMMP1* gene silencing affects developmental phenotypes in transgenic (Trans) rice compared with the non-transgenic control (Cont) plant. (**aI**) Photograph shows smaller grain size in transgenic rice lines. Scale bar = 10 mm. (**aII**) Comparison of thousand seed weight (gram) in control and transgenic rice lines. (**b**) Photographs show the comparison of germination capability of intact and dehusked rice grains. Scale bar = 20 mm. (**cI**) Scanning electron micrographs show shorter anther length in transgenic rice lines. The images were visualized using Zeiss EVO 60 scanning electron microscope. (**cII**) Comparison of anther length in control and transgenic rice lines. (**dI**) Photographs show the variation of seedling growth in transgenic and control rice lines under the dark (D) and light (L) conditions. Scale bar = 55 mm. (**dII**) Comparison shows the shoot length is shorter in transgenic lines relative to the control under the dark (D) condition, but not significantly altered under the light (L) condition. In all bar graphs, data are expressed as mean ± SE of 3 set observations (each transgenic line) for (**aII**); as mean ± SE of 16 observations (randomly from all transgenic lines) for (**cII**); as mean ± SE of 4 observations (each transgenic line) for (**dII**). Data are subjected to Student’s *t*-test: *P < 0.05 for (**aII**), (**bII**) and (**cII**). There is no significant difference between non-transgenic control and transgenic plant grown under light. ns, not significant.

To find out the *loss-of-function* phenotype in the autologous host, transgenic rice lines were developed using the *Agrobacterium* binary plasmid containing the *OsMMP1* hairpin RNA-generating element driven by rice polyubiquitin promoter (Supplementary Fig. S13). Analysis revealed the decrease in grain size and thousand seed weight in OsMMP1 down-regulated rice lines (Fig. 5aI,aII). Germination of seeds showed certain interesting results. The dehusked transgenic grains germinated synchronously with dehusked control grains, but the intact (with husk) grains of transgenic lines had a slower rate of germination and growth (Fig. 5b). Moreover, the anther length in RNAi rice lines was found to be reduced significantly (Fig. 5cI,cII).

In another experiment, dehusked rice seeds of transgenic and control plants were grown under the light and dark conditions separately for seven days at 27 °C temperature. We observed that the shoot length of RNAi transgenic seedlings was shorter compared to the control under dark, whereas no significant difference was noted under the normal light condition (Fig. 5dI,dII). Thus, the results obtained from the gene silencing in autologous host corroborate the findings of gene expression in heterologous system, implying the pleiotropic roles of OsMMP1 in growth and development.

### OsMMP1-expressing tobacco lines are resistant but down-regulated rice lines are susceptible to cell wall synthesis inhibitor

**Figure 6:**
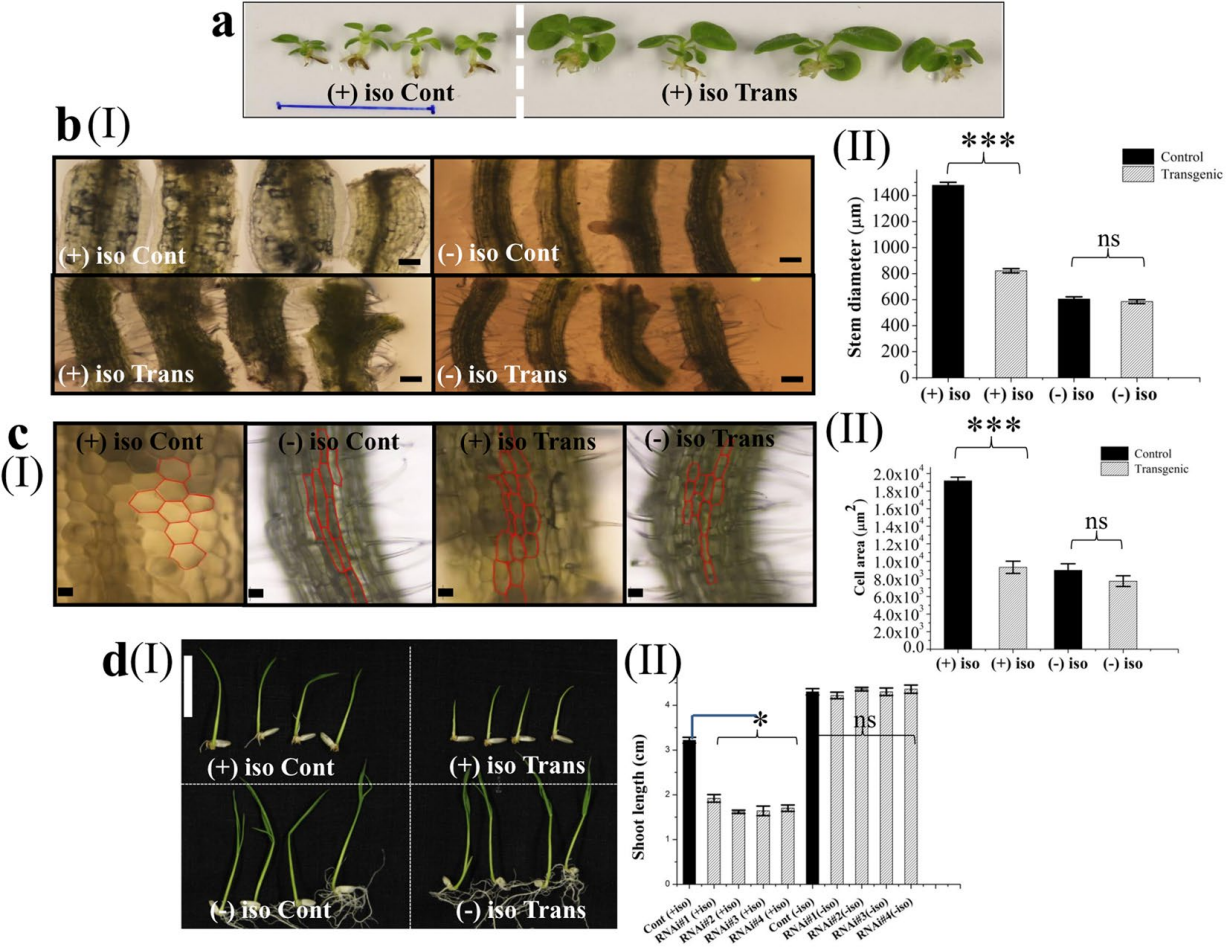
Effect of isoxaben, a cell wall synthesis inhibitor, on OsMMP1-expressing tobacco and OsMMP1 down-regulated rice plants. (**a**) Photograph exhibits the stunted growth of control (Cont) tobacco plants compared to transgenic (Trans) plants upon 1 nM isoxaben treatment (+iso). Scale bar = 25 mm. (**bI**) Micrographs show the stem diameter is increased significantly in the control tobacco plant compared with the transgenic lines upon isoxaben treatment (+iso). Scale bar = 300 µm. (**bII**) Comparison of stem diameter in control and transgenic tobacco plants with (+iso) and without (−iso) isoxaben treatment. (**cI**) Micrographs show the cell size is increased in control tobacco plant compared with the transgenic plant upon isoxaben treatment (+iso). Scale bar = 100 µm. (**cII**) Comparison of cell area (in µm^2^) in control and transgenic tobacco plants with (+iso) and without (−iso) isoxaben treatment. (**dI**) Photograph exhibits shorter shoot length in RNAi transgenic rice lines compared to the control upon 20 nM isoxaben treatment (+iso). Scale bar = 20 mm. (**dII**) Comparison of shoot length in control and transgenic rice plants with (+iso) and without (−iso) of isoxaben treatment. In all bar graphs, data are expressed as mean ± SE of 15 observations (each transgenic line) for (**bII**); as mean ± SE of 20 observations (each transgenic line) for (**cII**); as mean ± SE of 4 observations (each transgenic line) for (**dII**). Data are subjected to Student’s *t*-test: ***P < 0.001 for (**bII**) and (**cII**); *P < 0.05 for (**dII**). There is no significant difference between non-treated (−iso) control and transgenic. ns, not significant. Bright field micrographs of **bI** and **cI** were taken using Nikon ECLIPSE 50*i* upright microscope, and images were analysed with NLS-Elements D 3.2 software.

To understand whether the OsMMP1 protease has any role in cell wall-ECM modification, an experiment was designed to check the effect of isoxaben, a potent inhibitor of cellulose biosynthesis that produces the primary component of the cell wall. Tobacco seeds were germinated and grown on solid Murashige and Skoog (MS) medium supplemented with 1 nM isoxaben under the cycle of 16 h:8 h, light:dark with 750 lux and 60% humidity for 45 days. We found that transgenic lines expressing OsMMP1 were resistant to isoxaben, whereas control plant was critically affected by the treatment leading to stunted growth (Fig. 6a). The diameter of the stem was observed to increase significantly in the control plants compared to transgenic plants upon isoxaben application (Fig. 6bI,bII). Moreover, we noticed the radial growth with an enlarged area of epidermal cells in the control tobacco plant due to isoxaben treatment, whereas the transgenic plants were less prone to these changes caused by isoxaben (Fig. 6cI,cII).

Seeds from OsMMP1 down-regulated transgenic rice lines and control plants were germinated on solid MS media supplemented with 20 nM isoxaben. After seven days, we found that all RNAi lines grew shorter in height compared with the control (Fig. 6dI,dII), indicating transgenic rice plants are susceptible to isoxaben. On close observation, we noticed that the roots of transgenic rice seedlings failed to develop properly in comparison with the control plant upon isoxaben treatment (Fig. 6dI).

The contrasting phenotypic observations recorded from the OsMMP1-expressing tobacco and OsMMP1 down-regulated rice lines upon isoxaben treatment, further confirm that OsMMP1 plays a role in development and morphogenesis. We hypothesized that the compositional changes of cell wall constituents in transgenic lines could be the reason behind isoxaben responsiveness.

### OsMMP1 expression promotes cell wall modification in transgenic tobacco by enhancing cellulose and callose deposition

**Figure 7:**
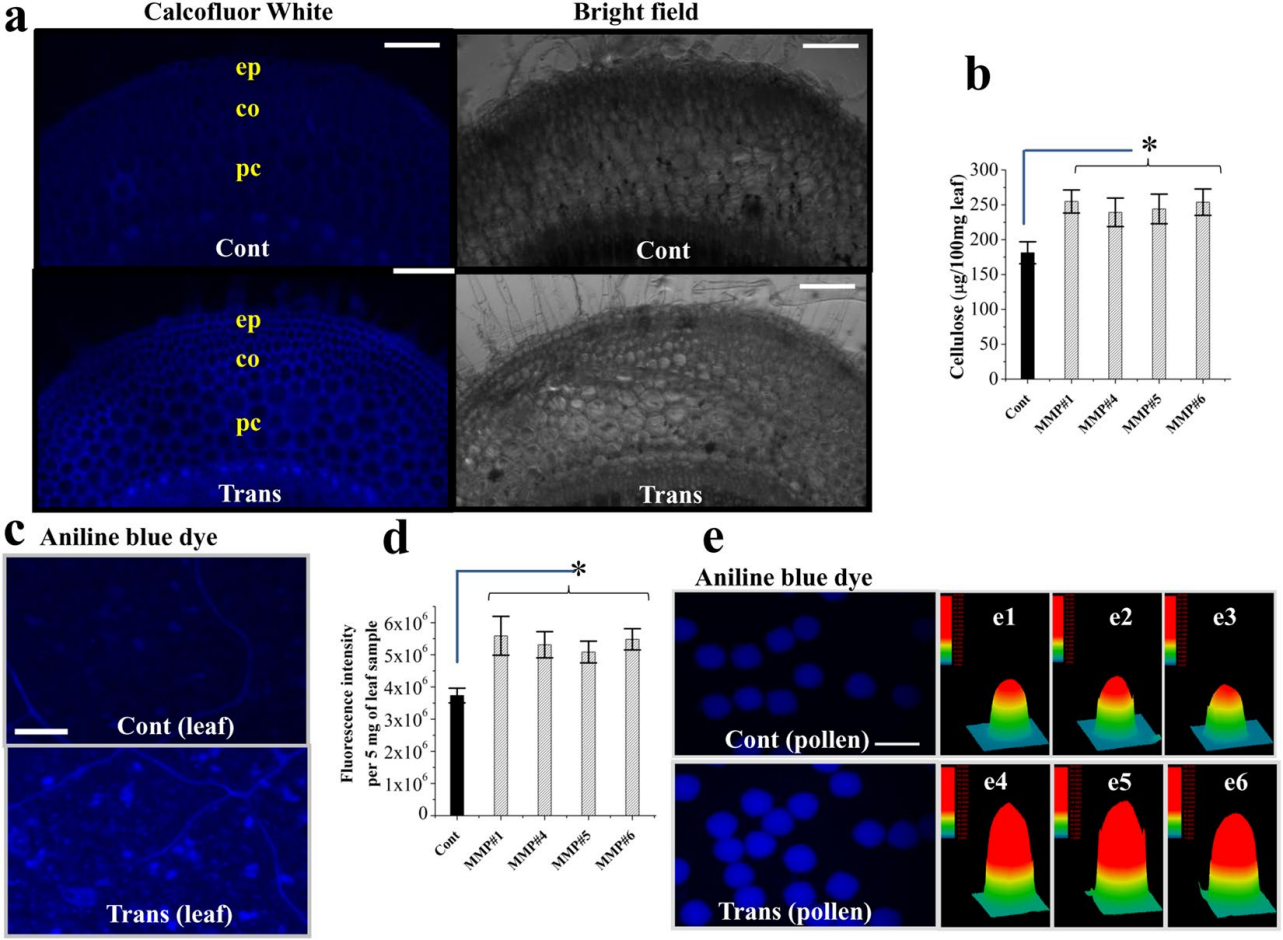
Qualitative and quantitative analyses of two cell wall constituents - cellulose and callose in transgenic (Trans) and non-transgenic control (Cont) tobacco plants. (**a**) Fluorescence micrographs show higher cellulose deposition in transgenic tobacco lines as revealed by Calcofluor White staining of stem cross sections. ep, epidermis; co, collenchyma; pc, parenchyma. Scale bar = 200 µm. (**b**) Comparison of the cellulose content (measured by anthrone test) in leaf tissue of fully grown control and transgenic plants. (**c**) Fluorescence micrographs exhibit higher callose deposition in transgenic lines as revealed by aniline blue staining of leaf samples. Scale bar = 100 µm. (**d**) Comparison of the callose content (fluorimetric quantification) in the leaf sample of fully grown control and transgenic plants. (**e**) Fluorescence micrographs show higher callose deposition in pollens of transgenic lines after aniline blue staining. Scale bar = 50 µm. (**e1–3** and **e4–6**) 3D surface plot images (by Image-Pro DISCOVERY software) of pollens represent fluorescence intensity of individual pollen after aniline blue staining. Fluorescence intensity is indicated by different color and height in the 3D surface plot. Fluorescence intensity is higher in transgenic pollens compared to control pollens. In all bar graphs, data are expressed as mean ± SE of 8 observations (each transgenic line) for (**b**) and as mean ± SE of 3 observations (each transgenic line) for (**d**). Data are subjected to Student’s *t*-test: *P < 0.05 for (**b**) and (**d**). For each sample type, micrograph was taken with the same exposure setting.

To test our hypothesis of altered cell wall constituents, we examined the cellular content of two important β-glucans, i.e., cellulose and callose, as their biosynthesis is shared by UDP-glucose as the common substrate. Calcofluor White staining revealed a higher level of cellulose deposition in stem cross-sections of transgenic tobacco lines compared to the control plant (Fig. 7a). The anthrone test was carried out to estimate the cellulose content. The result showed that the cellulose content in leaf tissue was increased by ~39% in transgenic tobacco lines compared with the control (Fig. 7b).

Similarly, callose deposition was assessed qualitatively and quantitatively. Leaf samples stained with aniline blue showed intense fluorescence signal in the cells of transgenic tobacco lines compared to the control plant (Fig. 7c). The total callose content in leaf tissue was quantified through fluorimetry, and the analysis revealed that the mean fluorescence intensity was increased by 43% in transgenic tobacco lines (Fig. 7d). Thus, the finding confirms that the transgenic lines synthesize higher amount of callose in comparison with the non-transgenic plants. Further analysis revealed that pollens of the transgenic lines accumulated more callose than the control ones (Fig. 7e).

### OsMMP1 expression affects the symplastic and apoplastic connections of tissues in transgenic tobacco

**Figure 8:**
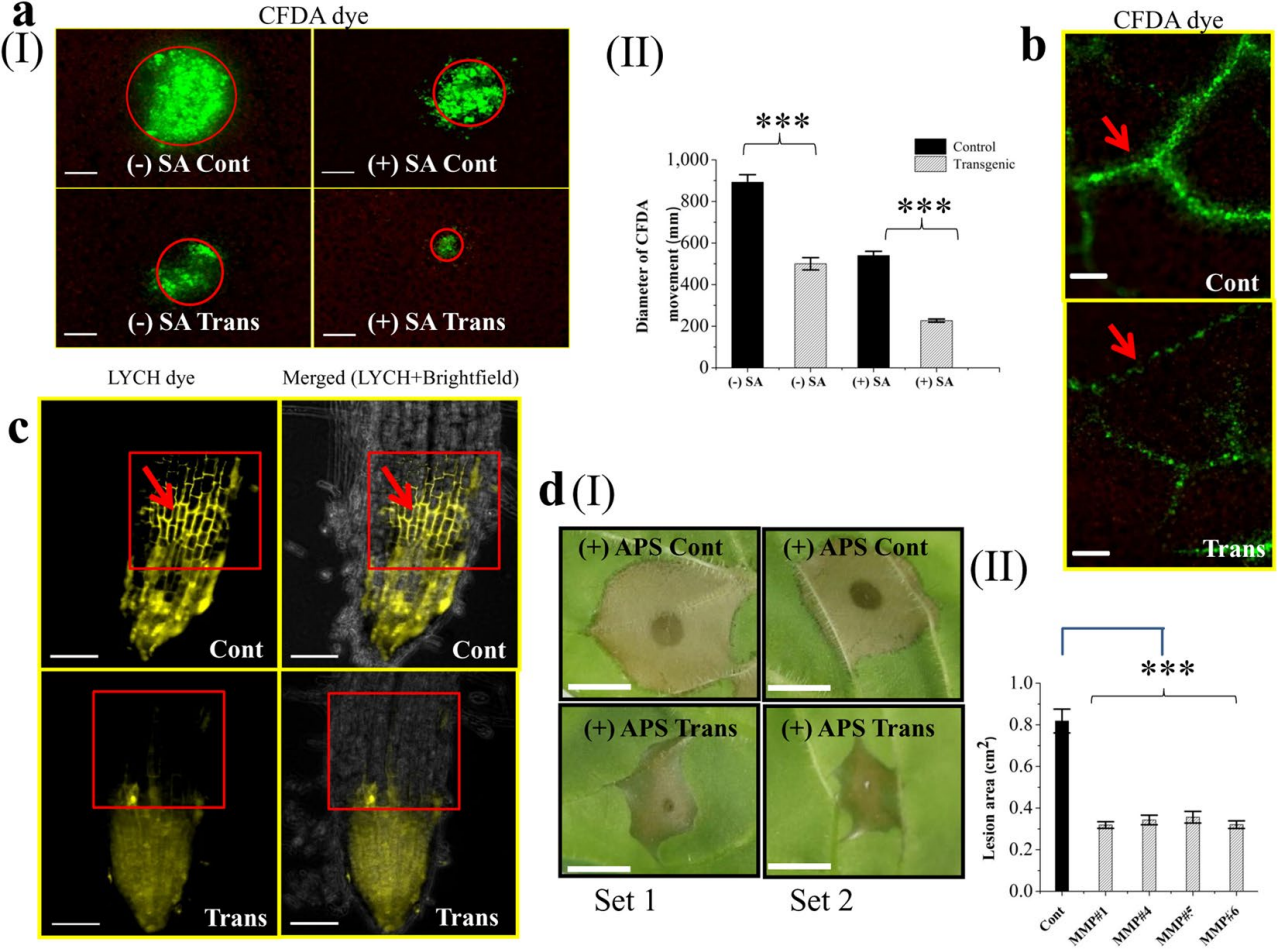
Monitoring symplastic transport, apoplastic transport, and necrosis spreading in transgenic (Trans) and non-transgenic control (Cont) tobacco plants. (**aI**) Micrographs of Drop-AND-See (DANS) experiment show the symplastic non-vascular movement of CFDA dye in tobacco leaf with (+) or without (−) salicylic acid (SA) treatment for 12 h. Fluorescence signal from CFDA was visualized by epifluorescence microscopy. Symplastic movement of CFDA dye is retarded in transgenic leaf without (−) SA application. Moreover, the transgenic sample shows further retardation of CFDA movement upon SA treatment. The circle shows the zone of CFDA dye spreading. Scale bar = 200 µm. (**aII**) Comparison of the extent of CFDA movement (measured in terms of the diameter of dye diffusion area) in control and transgenic plants with (+) or without (−) SA treatment. (**b**) Micrograph shows the symplastic vascular movement of CFDA dye in tobacco leaf. Transgenic leaf exhibits lesser translocation and symplastic unloading of dye from the vain to the adjacent area. Arrow denotes the leaf veins which provide vascular transport system. Scale bar = 200 µm. (**c**) Micrographs show the apoplastic movement of Lucifer Yellow CH (LYCH), potassium salt dye in tobacco root. Apoplastic movement of the LYCH dye is greater in the control root compared to transgenic root. Arrow indicates the upward movement of dye (indicated by a box) along the root axis through the apoplastic region of the cells. Scale bar = 200 µm. (**dI**) Photographs of two representative sets show the development of a larger lesion area in control leaf, but a smaller lesion area in transgenic leaf due to APS application. APS was spotted on leaf abaxial side to generate reactive oxygen species-signaling. Scale bar = 5 mm. (**dII**) Comparison of the lesion area in leaf samples of the control and transgenic plants. In all bar graphs, data are expressed as mean ± SE of 5 observations (each transgenic line) for (**aII**) and as mean ± SE of 10 observations (each transgenic line) for (**dII**). Data are subjected to Student’s *t*-test: ***P < 0.001.

Since the cell wall composition regulates the symplastic and apoplastic pathways, we examined these connections via the specific nature of the dye. To understand the non-vascular symplastic movement, plasmodesmata (PD) permeability was checked with carboxyfluorescin diacetate (CFDA) dye following the Drop-And-See (DANS) method. We observed that the CFDA diffusion was affected in the transgenic tobacco lines, as the dye traversed less distance (diameter of diffusion) compared to the control plant (Fig. 8aI,aII). To monitor the salicylic acid (SA)-induced PD permeability, the DANS assay was conducted using SA-treated leaf samples. Interestingly, we observed that the CFDA diffusion was significantly reduced in both SA-treated transgenic and control tobacco leaves compared to the untreated plant; however, the transgenic sample was affected more (Fig. 8aI,aII). Additionally, vascular symplastic movement was also investigated by applying the CFDA dye into the vascular system near the leaf petiole through an incision followed by incubation for 10 min. The CFDA dye traversed less efficiently in a transgenic sample through vascular transport with lesser dye load, whereas the control leaf was highly efficient in dye transporting and phloem unloading (Fig. 8b).

To examine the apoplastic convective system, 0.5% Lucifer Yellow CH (LYCH) dye was spotted on the root tips, and incubated 1 h for absorption and transportation. It was observed that the LYCH dye migrated lesser distance along the root axis through the apoplastic region in the transgenic plant compared with the control (Fig. 8c). The finding implies that the apoplastic movement is also hampered in OsMMP1-expressing tobacco lines, similar to the symplastic transport.

To understand the effect of restricted symplastic and apoplastic movements under stress stimuli, abaxial side of tobacco leaf samples was spotted with 1% ammonium persulfate (APS), an agent known to cause oxidative stress. Burnt-lesion area was observed in both control and transgenic leaves after 24 h of APS treatment. However, the lesion area in transgenic leaf samples was smaller compared to the control (Fig. 8dI,dII), suggesting that the activity of OsMMP1 limits the boundary of cell death.

## Discussion

We have characterized the *OsMMP1* gene product from an *indica* rice cultivar to understand how plant MMP maintains cell wall integrity, influences various developmental changes, and provides defence mechanism.

Model-based structural analysis has disclosed that the OsMMP1 protein is similar to human MMPs with respect to cofactor orientation in the catalytic domain and predicted to have protease activity (Fig. 1). Interestingly, unlike these MMPs, the OsMMP1 protease does not possess a hemopexin-fold domain. It is worth mentioning here that the rice and other plant species have a separate gene that encodes hemopexin-fold domain containing protein^22^, which does not show any protease activity. The protease activity of OsMMP1 was confirmed by the digestion of BSA and gelatin using the bacterially expressed recombinant OsMMP1 (Fig. 1). The findings imply that the structure dependent proteolytic activities of MMP proteins are evolutionary conserved in different species; however, they are involved in diverse functions.

The function of a gene product is only meaningful when the tissue specificity of the gene expression is known. For this purpose, firstly the *OsMMP1* transcript level was examined at the early developmental stages of the rice plant; and secondly, the promoter activity of the gene was analyzed in rice and tobacco systems using the *GUS* and *GFP* reporter genes, respectively. Expression pattern of the endogenous *OsMMP1* gene (Fig. 2) suggests its critical roles in plant growth and development. Presence of several common *cis*-regulatory elements of many housekeeping and inducible genes within the *OsMMP1* promoter (Supplementary Fig. S6) along with the findings of GUS reporter expressions in rice root, stem, leaf, flower bud, anther, and gynoecium (Fig. 2) provide vital information that the gene is active in most plant tissues. Similarly, the spatio-temporal regulation of the heterologously expressed *OsMMP1* promoter-*GFP* reporter in vegetative (radicle and stem) and reproductive (stigma and anther) tissues of tobacco (Supplementary Fig. S9) justifies that OsMMP1 is associated with different aspects of plant development. Previous reports have documented variable expressions of plant MMPs in different tissues and stages. The expression pattern of the Pta1-MMP of loblolly pine was found to differ at different developmental stages even at tissue specification^23^. Expressions of Arabidopsis MMPs were detected in roots, leaves, stems, and flowers^16^. All these studies including ours signify that these proteolytic enzymes are omnipresent in a given plant species.

Sub-cellular localization in OsMMP1-expressing transgenic tobacco lines revealed the presence of OsMMP1 in the plasma membrane (Fig. 3). According to *in-silico* analyses (PSORT, InterPro, and Big-PI plant predictor), the OsMMP1 protease has a transmembrane domain and a glycosylphosphatidylinositol (GPI)-binding domain. Therefore, like the GPI-anchored proteins^24,25^, OsMMP1 is expected to arrive first at the plasma membrane and subsequently is released into the cell wall region. The further experimental evidence is required to verify this hypothesis.

Different developmental changes, like early lateral root formation, longer primary root length, shorter hypocotyl length and lesser seed weight observed in OsMMP1-expressing tobacco seedlings (Fig. 4) imply that the OsMMP1 activity is crucial for the plant development and growth. A cuticle layer with increased thickness was observed in OsMMP1-expressing transgenic plants (Fig. 4d), resulting in slower cuticular transpiration (Fig. 4e). The cuticle is an external layer of the aerial epidermis, which provides the first level of defence against phytopathogen including protection against stress and desiccation^26^. Therefore, it is anticipated that OsMMP1 may play a role in defence mechanism; however, this needs further experimental validation.

Analysis of rice OsMMP1 RNAi lines also revealed the association of this OsMMP1 with different developmental aspects, where smaller grain size and shorter anther length are prominent pieces of evidence (Fig. 5). Light is the essential factor for plant growth and development. *In-silico* analysis of the 2 kb *OsMMP1* promoter unveiled the predominant (42%) occurrence of light responsive elements (Supplementary Fig. S6). Based upon this prediction, when rice seeds were germinated and grown for seven days in dark, we found that the shoot length is decreased markedly in RNAi lines compared to control plant (Fig. 5d). The exact mechanism of OsMMP1 in relation to light is still unclear, but we speculate that the protease activity of OsMMP1 might be responsible for activation or inactivation of some other light responsive proteins.

Therefore, the pleiotropic roles played by OsMMP1 are evidenced from the phenotypes of OsMMP1-expressing heterologous tobacco and OsMMP1 down-regulated autologous rice plants. Previous reports have also documented pleiotropic functions by other rice gene products, such as OsGRF4 in the context of grain shape, panicle length, and seed shattering^27^; and AFD1 in relation to plant height, floral development, and grain yield^28^.

We have observed that the OsMMP1 protein is localized in the plasma membrane and hypothesized to play a role in the maintenance of cell wall integrity. Therefore, an inhibitor was deployed to disturb the normal cell wall formation. Isoxaben is known to have the cellulose synthase inhibition effect^29^. We have observed that the tobacco plants expressing OsMMP1 are resistance to isoxaben, while the control plant is susceptible (Fig. 6). Isoxaben induces radial tissue swelling in Arabidopsis^30^ as well as reduces cellulose content in tobacco BY21 cells^31^. We have noticed that upon isoxaben treatment, the typically elongated cells of control tobacco plant show radial swelling, leading to significantly increased cell area compared with the transgenic plants (Fig. 6c). Biochemical analysis revealed that the higher cellulose content in transgenic tobacco plant (Fig. 7a,b) is the reason behind its more resilience against isoxaben, as cellulose provides mechanical strength to the dividing as well as elongated cells^32^. Interestingly, in a similar type of experiment using RNAi rice lines, we have found that the transgenic rice seedlings are more susceptible to isoxaben (Fig. 6d). Thus, the results from two different approaches of transgenesis (*gain-of-function* and *loss-of-function*) confirm that the OsMMP1 activity is associated with cell wall modification. However, the exact molecular mechanism involved in the enhancement of cellulose content through the functional activity of OsMMP1 is yet to be identified.

The symplastic and apoplastic pathways are critical for development, defence and survival of the plant species; and the cell wall constituents directly modulate these pathways. We have documented the retarded symplastic movement in OsMMP1-expressing tobacco plants that exhibit the reduced PD permeability of CFDA dye (Fig. 8); and this could be attributed to the higher level of callose deposition (Fig. 7c,d,e). Callose exists in the specialized cell wall and plays important roles in plant morphogenesis, development, and growth through its biogenesis and degradation^33^. The regulation of callose deposition and PD permeability has different impact on plant physiology^34–36^. PD regulates the trafficking of nutrients and signal molecules, and modulates intercellular coordination of biochemical and physiological processes by allowing direct cytoplasmic connection^37,38^. Though the manipulation of PD passageway is not entirely known, it is clear that the transportation of environmentally induced macromolecules could alter the aperture of the PD in regional and/or whole plant coordination^39,40^. The degree of callose deposition influences the size exclusion limit of PD, and high callose deposition at the neck of PD reduces the PD trafficking system^36,41^. The phenotype of early root emergence (Fig. 4a) in OsMMP1-expressing tobacco lines could be due to the higher degree of callose deposition in PD, which accelerates the restriction in symplastic connectivity of primordial cell. The restricted boundary of root primordial cell maintains the osmotic potential that facilitates organ emergence^42^.

In a parallel experiment, we have shown how SA controls the symplastic movement of CFDA dye by regulating PD permeability, particularly in transgenic plants (Fig. 8a). SA is known to promote PD closure by increasing callose deposition^43^. It has been reported that SA can restrict PD permeability by upregulating a PD inhibitor PDLP5 during an immune response^44^. Hence, the reason for the more restricted movement of CFDA dye in transgenic tobacco upon SA application is due to the increased level of callose deposition as a consequence of the dual effect of OsMMP1 activity and SA induction. Similarly, the obstructed apoplastic movement of LYCH dye (Fig. 8c) in OsMMP1-expressing transgenic plants is evidently correlated with the cell wall modification.

Callose deposition is responsive to biotic and abiotic stresses^45^. We have found that OsMMP1-expressing tobacco leaves are more resistant to the spread of necrosis caused by the oxidizing agent APS (Fig. 8d). This phenomenon could be easily linked to the enhanced callose accumulation and the modified cell wall with increased cellulose content, which together provide mechanical defence and might act as a diffusion barrier^46^. Moreover, callose restricts the toxic effect simply by blocking the cell to cell connection to reduce the spread of disease lesion in transgenic tobacco leaves. Mechanism of actual control of callose biosynthesis or cell wall modification due to environmental stress is not well established, but it is proved that stresses induce reactive oxygen species together with the accumulation of callose or cellulose^47,48^.

The present work shows a correlation between the OsMMP1 protease activity and β-glucan biosynthesis, as the OsMMP1-expressing tobacco lines accumulate higher content of callose and cellulose. Studies have reported that different degree of proteolysis is responsible for controlling the activity of 1,3-β-glucan synthase and 1,4-β-glucan synthase in fungal and plant species^49–52^. Although the exact molecular mechanism is not yet known, we hypothesize that the protease activity of OsMMP1 may play a role in activation of the β-glucan synthase.

In conclusion, we document for the first time the connection of a plasma membrane-localized MMP activity with plant growth, organ differentiation, and development in relation to cell wall modification. Considering the importance of spatio-temporal modulation of the cell wall-ECM structure and function throughout the lifespan of a plant, this study opens up further work on the biotechnological aspect of cell wall-ECM research with plant MMPs.

## Materials and Methods

### PCR-mediated cloning of the *OsMMP1* CDS, promoter and partial 3′-UTR region

To identify the MMP-like protein(s) in *indica* rice, BLASTP analysis was performed by selecting the non-redundant protein sequences of *Oryza sativa* (*indica* cultivar-group) available in NCBI database with the reported tobacco (*Nicotiana tabaccum*) NtMMP1 protein (GenBank: ABF58910)^20^ as a query. Among three significant hits (Supplementary Figs S1–S3), the putative rice MMP (EAY87473.1) having a maximum score of 315 with the highest amino acid identity of 50% and query coverage of 80% was designated as OsMMP1. The *OsMMP1* CDS and it’s predicted ~2 kb promoter region were PCR amplified from the genomic DNA (the CDS has no intron) of the *indica* rice cultivar IR64. The partial 3′-UTR region was amplified using OneStep RT (reverse transcriptase)-PCR kit (Qiagen). Forward and reverse primer pairs specific for CDS, promoter, and 3′-UTR of *OsMMP1* were designed (Supplementary Table S1) based on the sequence (NM_001054610) available at NCBI. To facilitate directional cloning in future, the forward and reverse primers were designed with the incorporated restriction enzyme sites of *Bam*HI and *Sac*I for the CDS, *Pst*I and *Bam*HI for the promoter, and *Bam*HI and *Sac*I for the 3′-UTR region. The respective PCR-amplified DNA fragments were cloned in pTZ57R/T plasmid (insTAclone, Fermentas). After sequencing the insert DNA fragments from a few selected clones, the nucleotide sequences were used for bioinformatics analysis. The nucleotide sequence comprising of *OsMMP1* CDS, its promoter and partial 3′-UTR has been deposited in GenBank (KY575874).

### Sequence analyses and tertiary structure prediction of OsMMP1

Clustal Omega^53^ was used for sequence alignment of OsMMP1 with *Arabidopsis thaliana* At1-MMP, *Glycine max* SMEP1, *Cucumis sativus* Cs1-MMP2, *Solanum lycopersicum* SL2-MMP and SL3-MMP, and *Nicotiana tabacum* Nt MMP1. The tertiary structure of OsMMP1 was predicted by using PHYRE 2^54^. The predicted model was evaluated through structure assessment tools, such as Ramachandran plot, VERIFY3D, and ERRAT (https://services.mbi.ucla.edu/SAVES/). PSORT^55^ and InterPro^56^ analyses were used to predict the presence of transmembrane domain in OsMMP1. Big-PI plant predictor^24^ was used to detect the presence of glycosylphosphatidylinositol (GPI)-modification site.

### Expression of recombinant OsMMP1 protein in *Escherichia coli*, purification and protease assay

The near full-length CDS of *OsMMP1* containing the amino acid sequence from 29^th^ to 323^rd^ was expressed in *E*. *coli* using the T7 polymerase-promoter system followed by purification of the His-tagged protein through Ni-NTA chromatography (Supplementary Methods). The refolding of insoluble recombinant OsMMP1 (rOsMMP1) protein was carried out using 6 M guanidine hydrochloride for subsequent proteolytic activity assay with two different proteins- BSA and gelatin as substrates (Supplementary Methods).

### Preparation of transgene constructs for rice transformation

For preparing the OsMMP1 promoter-GUS reporter gene construct, the 2 kb putative promoter region of the gene was sub-cloned in the pCAMBIA1391Z plasmid (Cambia, Australia) in *Pst*I-*Bam*HI orientation, at the upstream of the *gusA* CDS having the catalase intron. The prepared recombinant binary plasmid was denoted as pCAM::proMMP1/*GUS*/NOS (Supplementary Fig. S7).

For RNAi-mediated silencing construct, firstly a hairpin (hp) RNA forming hp-MMP1 DNA segment was prepared using a 156 bp inverted repeat of the partial 3′-UTR fragment of *OsMMP1* flanking the 191 bp arbitrary linker DNA. Next, the hp-MMP1 DNA segment was placed under the rice polyubiquitin 1 (Rubq 1) gene promoter (AY785814) in pCAMBIA1300 to develop the recombinant binary plasmid pCAM:: Rubq*/hp-MMP1/*NOS (Supplementary Fig. S13). The detail of this cloning procedure is described in Supplementary Methods.

Two binary plasmids - pCAM::proMMP1/*GUS*/NOS and pCAM::Rubq*/hp-MMP1/*NOS were introduced separately into *Agrobacterium tumefaciens* strain EHA105. Independent transgenic rice lines were developed following *Agrobacterium*-mediated embryogenic callus tissue transformation^57,58^. Putative rice transformants were selected on MS medium containing 50 mg l^−1^ hygromycin (Duchefa, Biochemie).

### Preparation of transgene construct for tobacco transformation

For preparing the OsMMP1 promoter-GFP reporter gene construct, the 2 kb putative promoter of *OsMMP1* along with the *GFP* reporter gene was cloned in pCAMBIA1300 to develop the recombinant binary plasmid pCAM::proMMP1/*GFP*/NOS (Supplementary Fig. S8). The detail of this cloning procedure is described in Supplementary Methods.

For preparing the *OsMMP1* CDS expression construct, firstly the enhanced 2X CaMV35S promoter and NOS terminator were cloned in pCAMBIA1300 at the *Hin*dIII-*Bam*HI and *Sac*I-*Eco*R1 sites, respectively. Subsequently, the *OsMMP1* CDS was cloned at the *Bam*HI-*Sac*I sites to generate the recombinant plasmid pCAM::2X35 S/*OsMMP1*/NOS (Supplementary Fig. S10).

Two binary plasmids- pCAM::proMMP1/*GFP*/NOS and pCAM::2X35S/*OsMMP1*/NOS were introduced separately into *A*. *tumefaciens* strain LBA 4404 VirGN^54^D. Independent transgenic tobacco lines were developed following *Agrobacterium*-mediated leaf disc transformation^59^. Putative tobacco transformants were selected on MS medium containing 250 mg l^−1^ hygromycin (Duchefa, Biochemie).

### Screening of transformed rice and tobacco plants

Following hardening in the glasshouse, a few selected putative transgenic lines of rice and tobacco were subjected to PCR screening followed by Southern hybridization to confirm genomic integration of the respective transgene (Supplementary Methods). For each of the four transgene constructs, the following numbers of transgenic lines were developed: OsMMP1promoter:GUS in rice- nine, OsMMP1promoter:GFP in tobacco- 16, *OsMMP1* gene overexpression in tobacco- 20, and *OsMMP1* RNAi in rice-11. Finally, we selected four non-sibling independent stable (T3 generation) transgenic lines of each transgene for all kind of experiments.

### Plant growth condition

Rice and tobacco seeds were surface-sterilized with 2% mercuric chloride (Sigma) and 1% sodium hypochlorite (HiMedia) supplemented with the surfactant Tween-20 (Sigma) for 2 min 30 sec and 10 min, respectively. Seeds were then washed thoroughly four times with double distilled autoclaved water.

After removing excess water by blotting paper, rice seeds were placed on solid media (with 0.8% agar), and supplemented with or without 20 nM isoxaben (Sigma). Seeds were germinated and grown under 16 h:8 h, light:dark condition with 60% humidity, 28 °C temp, and 750 lux illumination inside the plant growth chamber (Environmental System, Daihan Labtech Co. Ltd.). For the light responsive experiment, rice seeds were placed on solid MS medium. Some plates were kept in dark and some were maintained at 16 h:8 h, light:dark condition. In both cases, the same temperature (28 °C temp) and light intensity (750 lux) were maintained, and observations were recorded after seven days.

Similarly, tobacco seeds were placed on solid MS media supplemented with or without 1 nM isoxaben (Sigma), and then incubated in dark for germination. Germinated tobacco seeds were grown under 16 h:8 h, light:dark condition with 60% humidity, 25 °C temp, and 750 lux illumination inside the plant growth chamber. Observations were recorded on a regular interval and documented after 45 days. For exogenous salicylic acid (SA) (Sigma) application, plants were sprayed with 500 µM SA and covered with polybag. The samples were subjected to DANS assay after 12 h.

### RNA extraction and quantitative reverse transcription PCR (qRT-PCR)

Total RNA was isolated from root and shoot tissues of *indica* rice cultivar IR64 at different developmental stages. Similarly, total RNA was also extracted from leaf tissue of RNAi rice lines and control plants. The details of the procedures of RNA isolation and qRT-PCR are described in Supplementary Methods.

### GUS assay

Histochemical GUS assay was performed with a minor modification of laboratory established protocol^60^. Rice tissues were incubated in GUS assay buffer (0.1 M sodium phosphate buffer pH 7.0, 0.1% Triton X-100, and 2 mM X-Gluc) at 37 °C for 16 h. Next, samples were washed thoroughly in 70% ethanol and kept in 70% ethanol until imaging.

### Immunohistochemistry of plant tissues

Sub-cellular localization of the heterologously expressed OsMMP1 protein in tobacco plant was detected following the reported technique^61,62^ with minor modification (Supplementary Methods).

### Nile Red staining for cuticle

Cuticle layer of tobacco tissue was detected with the reported Nile Red staining method^63^ (Supplementary Methods).

### Monitoring symplastic movement through CFDA staining

The DANS assay was employed^37^ to check the plasmodesmata (PD) permeability. A membrane permeable non-fluorescent dye, CFDA (Sigma) was used as a symplastic tracer. Once released into the cytoplasm, this non-fluorescent CFDA becomes fluorescent and membrane impermeable upon modification by cellular esterases. Only the modified CFDA could be transported via PD. The dye was spotted on the abaxial side of the tobacco leaf. After 5 min, drops were removed quickly with tissue paper and observed under the epifluorescence microscope. The SA-treated and untreated plant samples were also used in the DANS assay. Translocation through the vascular system was visualized by applying 10 µl of 1 mM CFDA dye at the cut portion of the petiole of the intact leaf. The result was observed after 10 min of incubation under an epifluorescence microscope (Olympus 1X 51, CFDA: blue excitation, barrier filter 520 nm; red autofluorescence: green excitation, barrier filter 590 nm).

### Monitoring apoplastic movement through LYCH staining

For monitoring the apoplastic transport system, tobacco plantlets were grown on solid MS media keeping the Petri plates vertically, and plantlets with only single primary root without secondary roots were selected for the experiment. A working solution of 0.5% Lucifer Yellow CH (LYCH), potassium salt (Invitrogen) was loaded on the root tip and incubated for 1 h. Subsequently, the root tip was observed under an epifluorescence microscope (Olympus 1X 51, blue excitation, barrier filter 520 nm).

### Qualitative and quantitative analysis of cellulose

Cellulose detection was carried out following the reported protocol^64^. Stem-cross sections were stained with 0.01% Calcofluor White (Sigma) for 30 seconds and then washed thoroughly. The samples were observed under the epifluorescence microscope (Olympus 1X 51, ultraviolet excitation, barrier filter 420 nm). For quantitative estimation of cellulose, standard anthrone assay protocol was followed^65^.

### Qualitative and quantitative analysis of callose

Qualitative detection of callose was performed through the aniline blue staining protocol reported previously^66^ with minor modification (Supplementary Methods). For quantitative estimation of callose, the fluorimetry-based reported protocol^67^ was followed with minor modification (Supplementary Methods).

### Statistical analyses of data

Data collection and observations were recorded from three or more biological samples of two or three experimental replicates. Specific numbers of data or observations are mentioned in the respective figure legends. Statistical analyses of the data obtained during the experiments were carried out using unpaired *t*-test with a two-tailed distribution. Changes in measurements were considered statistically significant at *P* < 0.05 and *P* < 0.001 for different experiments.

## Electronic supplementary material

Supplementary Information
