# Supplementary material for: Rice matrix metalloproteinase OsMMP1 plays pleiotropic roles in plant development and symplastic-apoplastic transport by modulating cellulose and callose depositions
Source: Sci Rep. 2018 Feb 9;8:2783. doi: 10.1038/s41598-018-20070-4 (PMC5807377; doi:10.1038/s41598-018-20070-4)
Supplement: Supplementary file 1 — Supplementary Information [file 41598_2018_20070_MOESM1_ESM.pdf]

SUPPLEMENTARY INFORMATION

Rice matrix metalloproteinase OsMMP1 plays pleiotropic roles in plant development and symplastic-apoplastic transport by modulating cellulose and callose depositions

Prabir Kumar Das, Rupam Biswas, Nazma Anjum, Amit Kumar Das, Mrinal K. Maiti*

Department of Biotechnology, Indian Institute of Technology Kharagpur, Kharagpur 721302, India

*Author for correspondence:

Mrinal K. Maiti

Telephone: 91-3222-283796

E-mail: maitimk@hijli.iitkgp.ernet.in, mrinalkmaiti@gmail.com

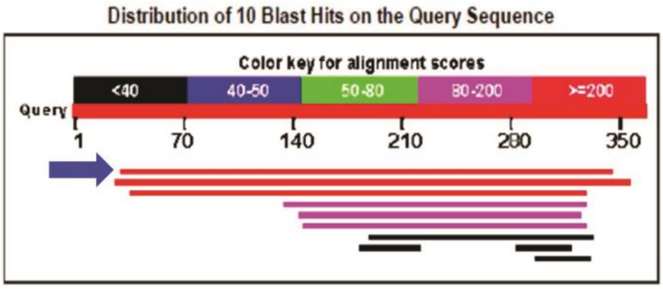

Description	Max score	Total score	Query cover	E value	Max ident	Accession
hypothetical protein OsI_08881 [Oryza sativa Indica Group]	315	315	85%	4e-104	50%	EAY87473.1
hypothetical protein OsI_22327 [Oryza sativa Indica Group]	297	297	89%	3e-97	48%	EAZ00310.1
hypothetical protein OsI_34615 [Oryza sativa Indica Group]	249	249	79%	7e-79	43%	EAY79487.1
hypothetical protein OsI_34618 [Oryza sativa Indica Group]	146	146	52%	2e-40	40%	EAY79490.1
hypothetical protein OsI_34616 [Oryza sativa Indica Group]	136	136	49%	9e-37	42%	EAY79488.1
hypothetical protein OsI_34617 [Oryza sativa Indica Group]	134	134	49%	7e-36	38%	EAY79489.1
hypothetical protein OsI_23479 [Oryza sativa Indica Group]	37.7	37.7	39%	0.012	27%	EEC80860.1
hypothetical protein OsI_16591 [Oryza sativa Indica Group]	29.3	29.3	9%	6.0	40%	EAY94806.1
hypothetical protein OsI_07517 [Oryza sativa Indica Group]	28.1	28.1	9%	9.1	37%	EAY86144.1
hypothetical protein OsI_24545 [Oryza sativa Indica Group]	28.5	28.5	10%	9.6	28%	EAZ02442.1

Supplementary Figure S1. Summary of BLASTP results. A few hypothetical MMP-like protein-coding sequences in *indica* rice (*Oryza sativa*) were identified through BLASTP search using the reported tobacco (*Nicotiana tabacum*) NtMMP1 protein (GenBank: protein accession no. ABF58910; cDNA accession no. DQ508374) as query. Among them, the putative rice MMP (EAY87473) having a maximum score of 315 with the highest amino acid identity of 50% and query coverage of 85% was designated as OsMMP1. The corresponding gene was cloned from the *indica* rice cultivar IR64; and after characterization, the gene sequence was submitted to GenBank (KY575874). The newly cloned gene encodes the OsMMP1 protein (AQU14357), which is identical with EAY87473.

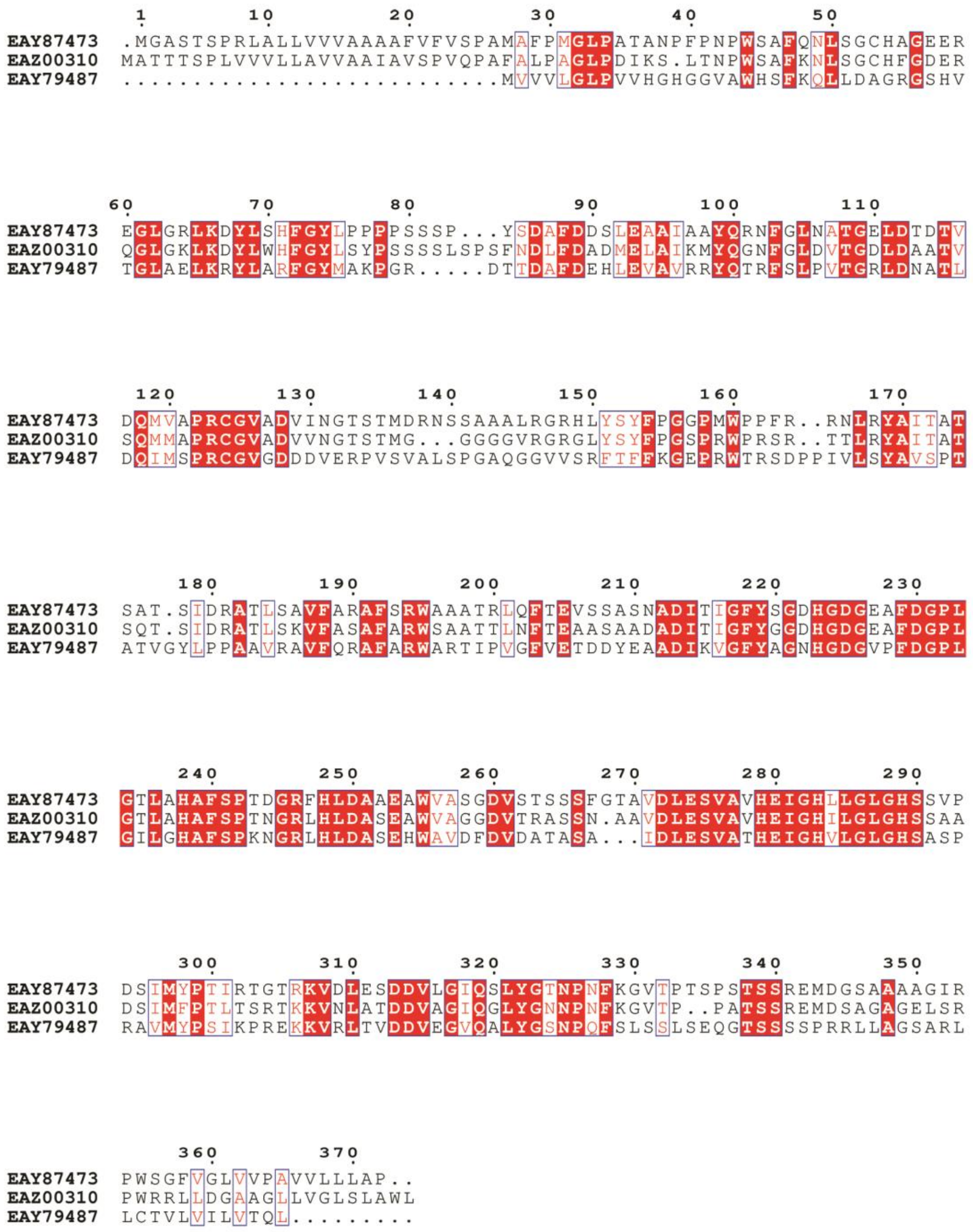

Supplementary Figure S2. Amino acid sequence alignment of three putative rice MMP proteins having high homology with tobacco NtMMP1 (GenBank: ABF58910). Three putative rice MMP proteins, i.e. EAY87473, EAZ00310 and EAY79487 identified through BLASTP were aligned in CLUSTALW (http://www.genome.jp/tools-bin/clustalw), and the final aligned sequences were depicted using ESPript 3.0 (http://espript.ibcp.fr/ESPript/cgi-bin/ESPript.cgi)[1]. The amino acid sequence of OsMMP1 protein (GenBank: AQU14357) is identical with EAY87473.

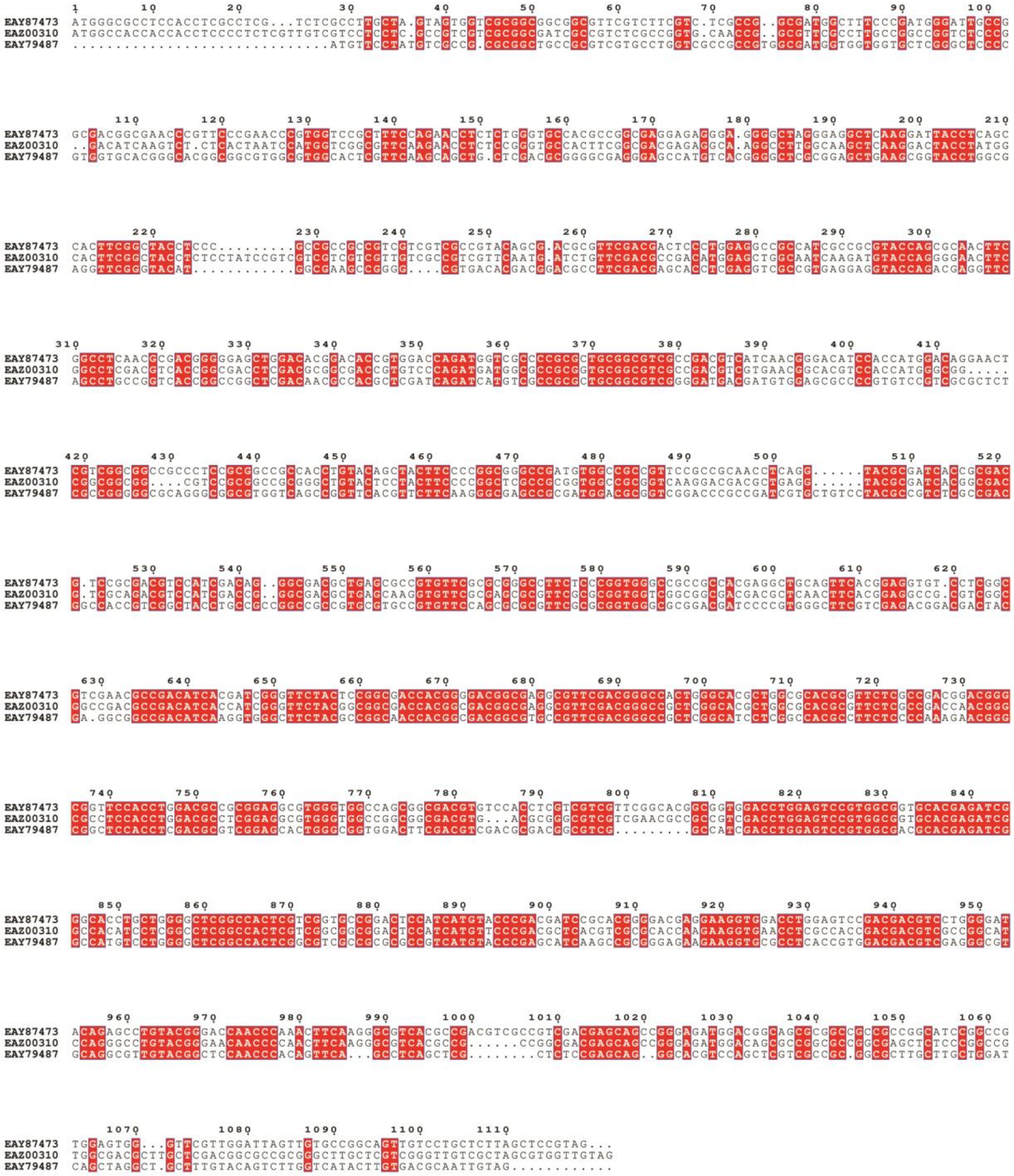

Supplementary Figure S3. Coding DNA sequence (CDS) alignment of three putative rice MMP proteins. The CDSs corresponding to EAY87473, EAZ00310 and EAY79487 were aligned in CLUSTALW (http://www.genome.jp/tools-bin/clustalw), and the final aligned sequences were depicted using ESPript 3.0 (http://espript.ibcp.fr/ESPript/cgi-bin/ESPript.cgi)[1]. The CDS of OsMMP1 gene (GenBank: KY575874) is identical with that of EAY87473.

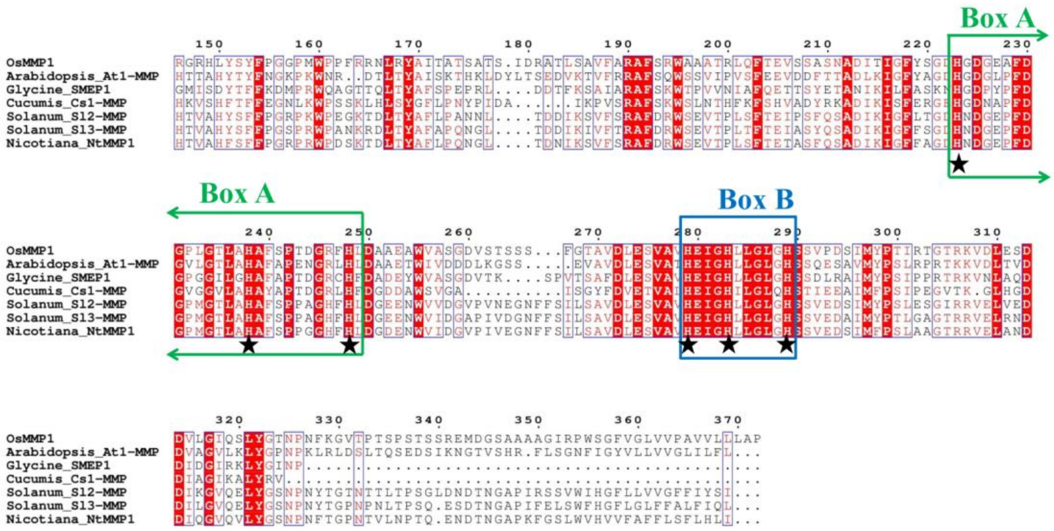

Supplementary Figure S4. Sequence alignment of OsMMP1 protein with other plant MMPs, displaying amino acid identity. The OsMMP1 (372 aa) was aligned with *Arabidopsis thaliana* At1-MMP (364 aa; GenBank, NM_117765), *Glycine max* SMEP1 (305 aa; GenBank, NM_001248606), *Cucumis sativus* Cs1-MMP2 (320 aa; GenBank, AJ133371), *Solanum lycopersicum* SL2-MMP and SL3-MMP (363 aa; GenBank, HE819182 and 367 aa; GenBank, HE819181) and *Nicotiana tabacum* NtMMP1 (365 aa; GenBank, DQ508374). Two Zn^{2+}-binding motifs are shown by Box A and Box B. Conserved His (H) residues involved in Zn^{2+} coordination are indicated by asterisk symbol. The amino acids close to the catalytic Zn^{2+}-binding His residues (Box B) are highly conserved compared to the structural Zn^{2+}-binding His residues (Box A). The accession numbers indicate the corresponding mRNA sequences. Sequences were aligned in CLUSTALW (http://www.genome.jp/tools-bin/clustalw), and the final aligned sequences were depicted with ESPript 3.0 (http://espript.ibcp.fr/ESPript/cgi-bin/ESPript.cgi)[1].

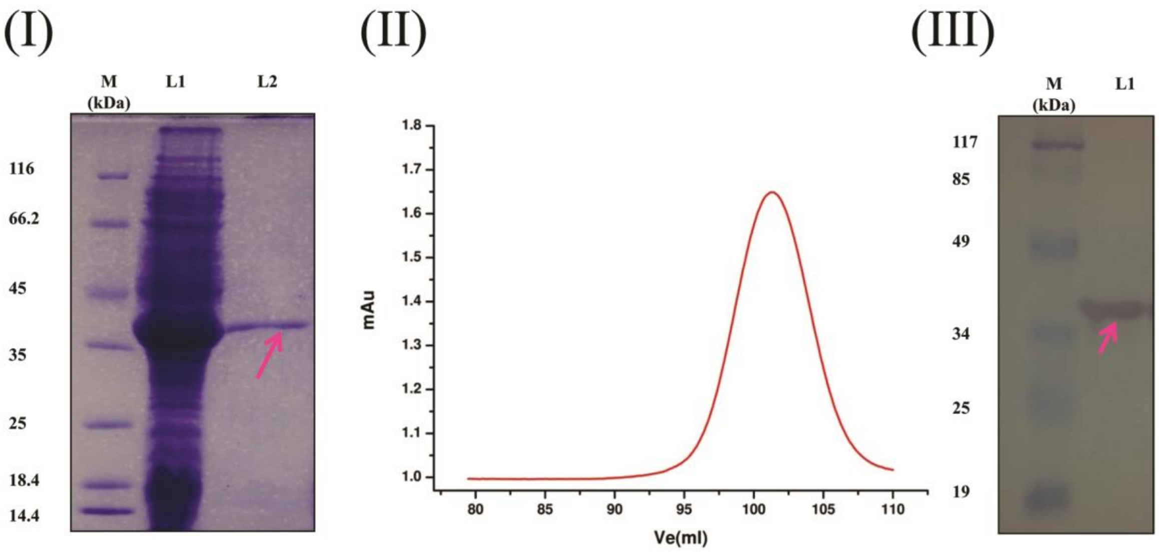

Supplementary Figure S5. Purification of the recombinant OsMMP1 (rOsMMP1) protein. (I) Bacterially expressed proteins resolved by 12% SDS-PAGE followed by Coomassie blue staining. Lanes M: protein molecular weight marker, L1: total protein from IPTG induced *E. coli* cells, L2: Ni-NTA purified rOsMMP1 protein (indicated by arrow). **(II)** The refolded rOsMMP1 protein was eluted in monomeric conformation as confirmed by size-exclusion chromatography (Superdex 200 prep-grade matrix in a Hiload 16/60 C column, GE Healthcare Biosciences). **(III)** Western-blot detection of the rOsMMP1protein (indicated by arrow) with custom made anti-OsMMP1 antibody. Full-length gel of (**I)** and blot of (**III)** are presented in Supplementary Fig. S14 and S15, respectively

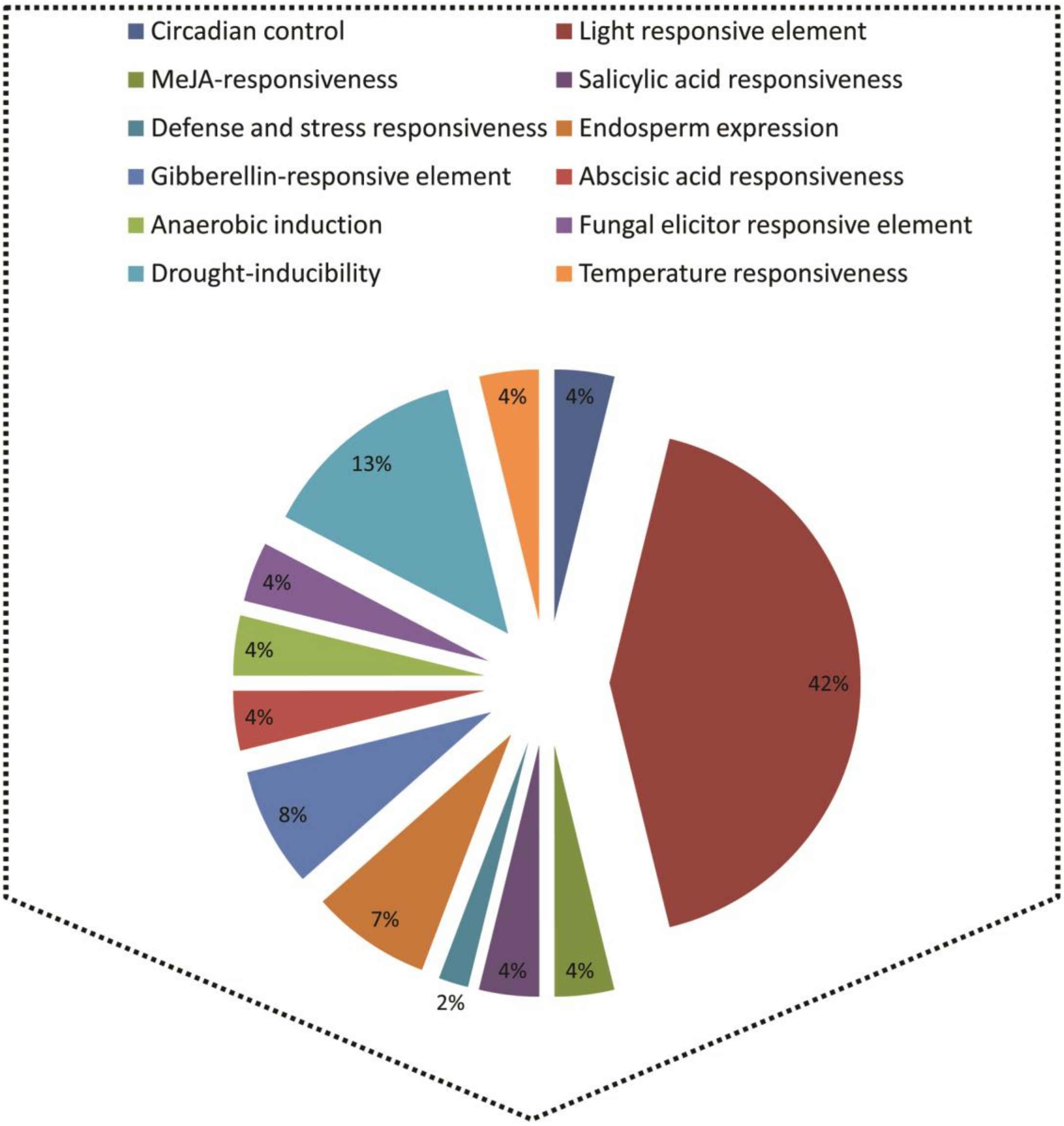

Supplementary Figure S6. *In-silico* analysis of the 2 kb putative *OsMMP1* promoter representing the presence of different *cis*-regulatory elements. PlantCARE softwere (http://bioinformatics.psb.ugent.be/webtools/plantcare/html/)[2] revealed the presence of several important *cis*-regulatory elements, like light responsive element (42%), temperature responsive (4%), endosperm expression element (7%), defence and stress responsiveness (2%), and circadian control element (4%). The pie chart was prepared in Microsoft Office using the PlantCARE analysis data.

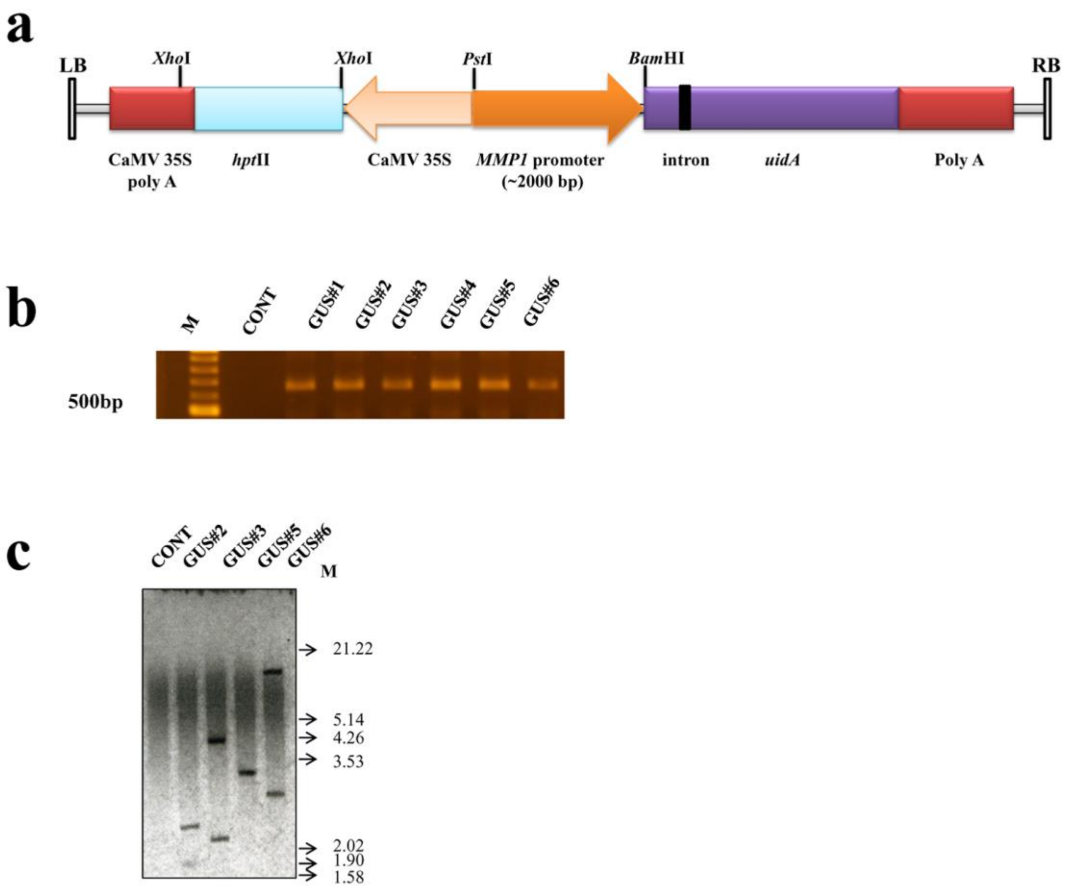

Supplementary Figure S7. Development of independent transgenic rice lines with *OsMMP1* promoter-*gusA* reporter gene construct. (a) Schematic diagram of the T-DNA part of pCAM::proMMP1/*GUS*/NOS construct prepared in the pCAMBIA1391Z binary plasmid. **(b)** Ethidium bromide stained 1% agarose gel showing PCR-based screening for the presence of a transgene-specific 650 bp DNA fragment (fusion part of the *OsMMP1* promoter and *gusA* or *uidA* gene) in transgenic rice lines (T3 generation) developed with the pCAM::proMMP1/*GUS*/NOS construct. Lanes M: 100 bp DNA ladder as molecular weight marker, CONT: untransformed control, GUS#1 to GUS#6: different transgenic lines. **(c)** Representative Southern blot showing the integration pattern of the transgene in T3 transgenic rice lines. Lanes CONT: untransformed control, GUS#2, #3, #5 and #6: four independent T3 transgenic lines, M: arrowheads indicate the position of *Eco*RI and *Hind*III digested lambda DNA as molecular weight marker.

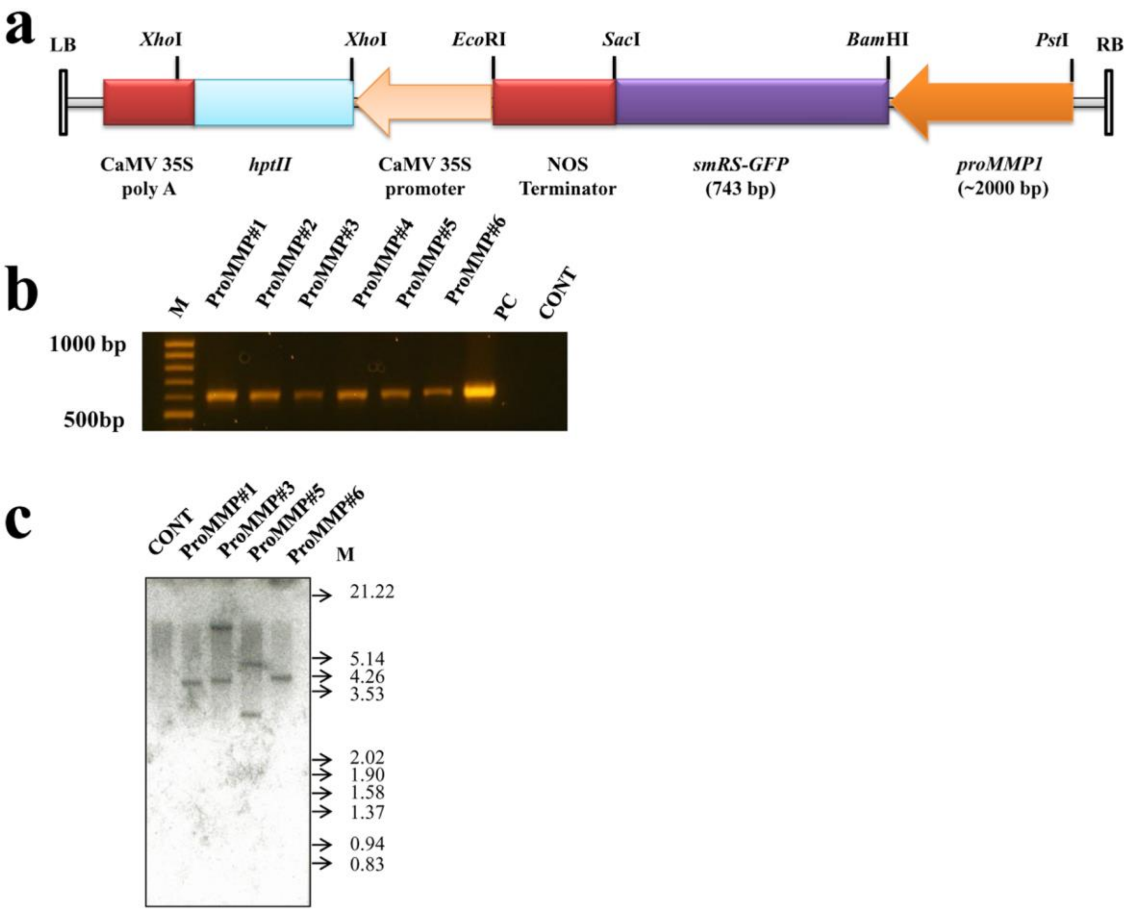

Supplementary Figure S8. Development of independent transgenic tobacco lines with *OsMMP1* promoter-*GFP* reporter gene construct. (a) Schematic diagram of the T-DNA part of pCAM::proMMP1/*GFP*/NOS construct prepared in the pCAMBIA1300 plasmid. **(b)** Ethidium bromide stained 1% agarose gel showing PCR-based screening for the presence of a transgene-specific 617 bp DNA fragment (fusion part of the promoter and *GFP* gene) in transgenic tobacco lines (T3 generation) developed with the pCAM::proMMP1/*GFP*/NOS construct. Lanes M: 100 bp DNA ladder as molecular weight marker, ProMMP#1 to ProMMP#6: different transgenic lines, PC: positive control, CONT: untransformed control. **(c)** Representative Southern blot showing the integration pattern of the transgene in T3 transgenic tobacco lines developed with the pCAM::proMMP1/*GFP*/NOS construct. Lanes CONT: untransformed control, ProMMP#1, #3, #5 and #6: four independent T3 transgenic lines, M: arrowheads indicate the position of *Eco*RI and *Hind*III digested lambda DNA as molecular weight marker.

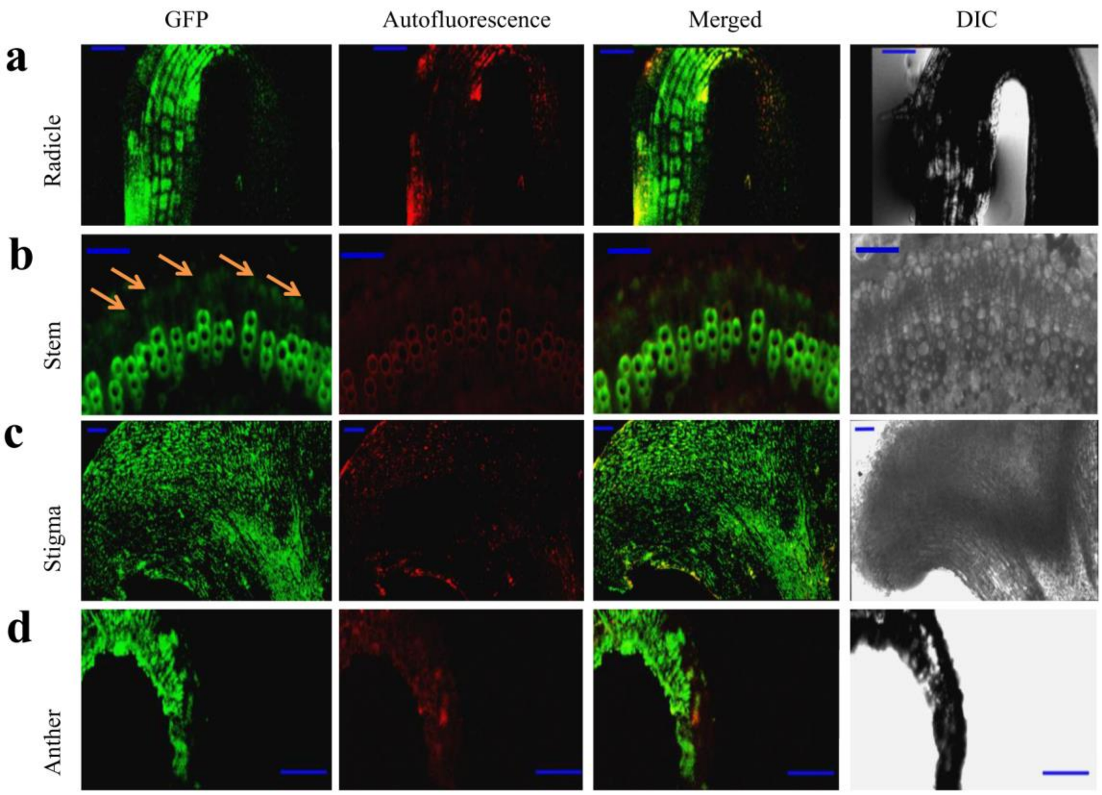

Supplementary Figure S9: The *OsMMP1* promoter-driven GFP expression in different tissue types of transgenic tobacco lines. Green fluorescence signal of GFP is noticed in **(a)** radicle of a germinating embryo, **(b)** stem cross section (indicated by arrows), **(c)** stigma cross section, and **(d)** anther transverse section. All images were taken with the confocal laser scanning microscope (Fluo View FV1000 confocal microscope, Olympus). The green GFP fluorescence and the red autofluorescence were captured with FITC and Alexaflour 568 filters, respectively. Scale bars for radical = 100 µm, stem = 50 µm, stigma = 100 µm, and anther = 100 µm.

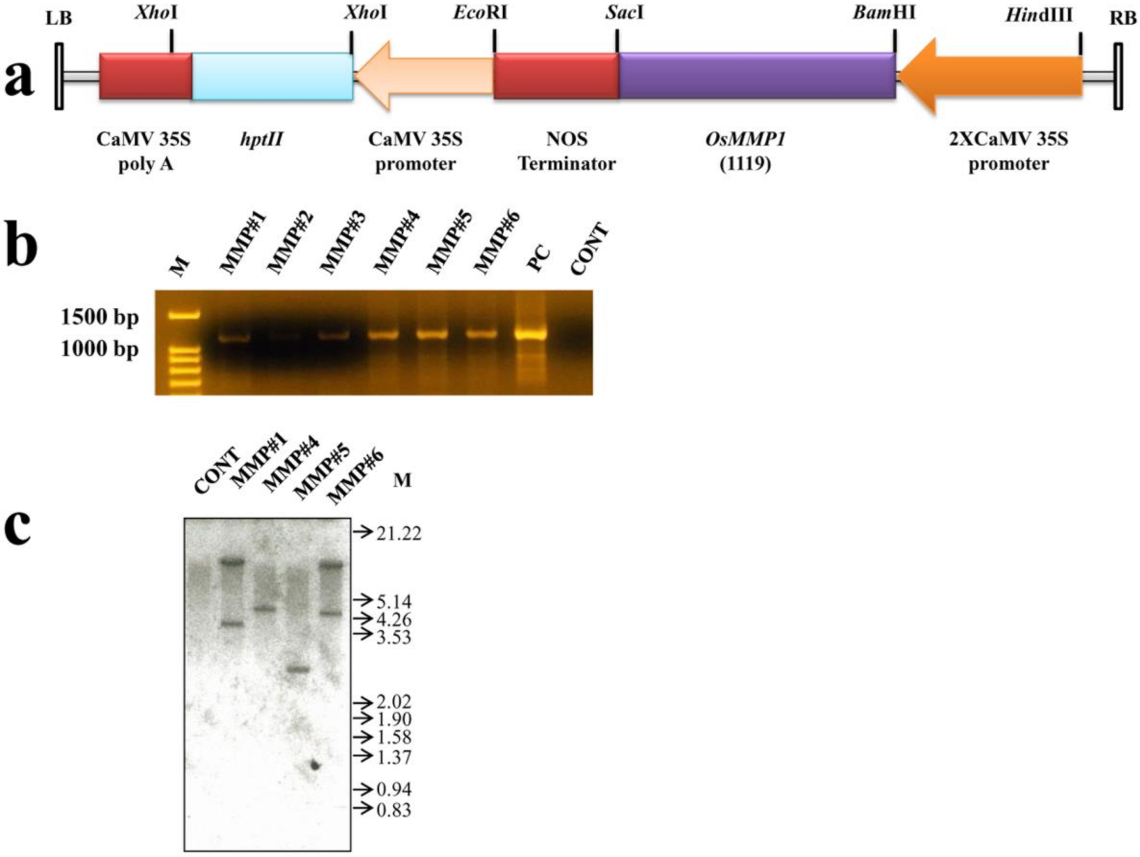

Supplementary Figure S10. Development of independent transgenic tobacco lines with 2XCaMV35S promoter-*OsMMP1* CDS expression construct. (a) Schematic diagram of the T-DNA part of pCAM*::*2X35S/*OsMMP1/*NOS construct prepared in the pCAMBIA1300 plasmid. **(b)** Ethidium bromide stained 1% agarose gel showing PCR-based screening for the presence of 1119 bp *OsMMP1*-specific amplicon in transgenic tobacco lines (T3 generation) developed with the pCAM::2X35S/*OsMMP1*/NOS construct. Lanes M: 100 bp DNA ladder as molecular weight marker, MMP#1 to MMP#6: different transgenic lines, PC: positive control, CONT: untransformed control. **(c)** Representative Southern blot showing the integration pattern of the transgene in T3 transgenic tobacco lines developed with the pCAM*::*2X35S*/OsMMP1/*NOS construct. Lanes CONT: untransformed control, MMP#1, #4, #5 and #6: four independent T3 transgenic lines, M: arrowheads indicate the position of *Eco*RI and *Hind*III digested lambda DNA as molecular weight marker.

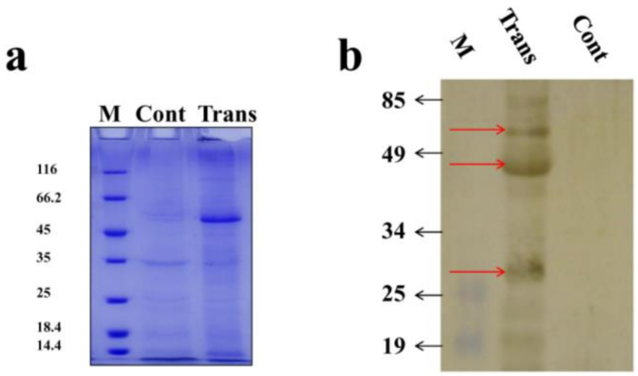

Supplementary Figure S11. Detection of recombinant OsMMP1 protein in transgenic tobacco. (a) Coomassie blue stained 12 % SDS-PAGE of total proteins (80 µg per lane) extracted from leaf sample of control (Cont) and OsMMP1-expressing tobacco plant (Trans). **(b)** Western blot of the protein sample (40 µg per lane) extracted from control and transgenic plant with anti-OsMMP1 antibody.. No signal detected in control lane (Cont) but three prominent bands detected in transgenic lane (Trans). Immunoblot analysis revealed that the OsMMP1 protein may exist in many forms in transgenic tobacco with masses near and greater than 49 kDa marker band. The calculated theoretical mass of the OsMMP1 (latent or inactive) protein without signal peptide is 36.3 kDa. The difference between the predicted and observed values probably reflects post-translational modification including glycosylation. The lower band near 25 kDa molecular weight most likely represents the processed and active form of OsMMP1, analogous to the reported tobacco NtMMP1.

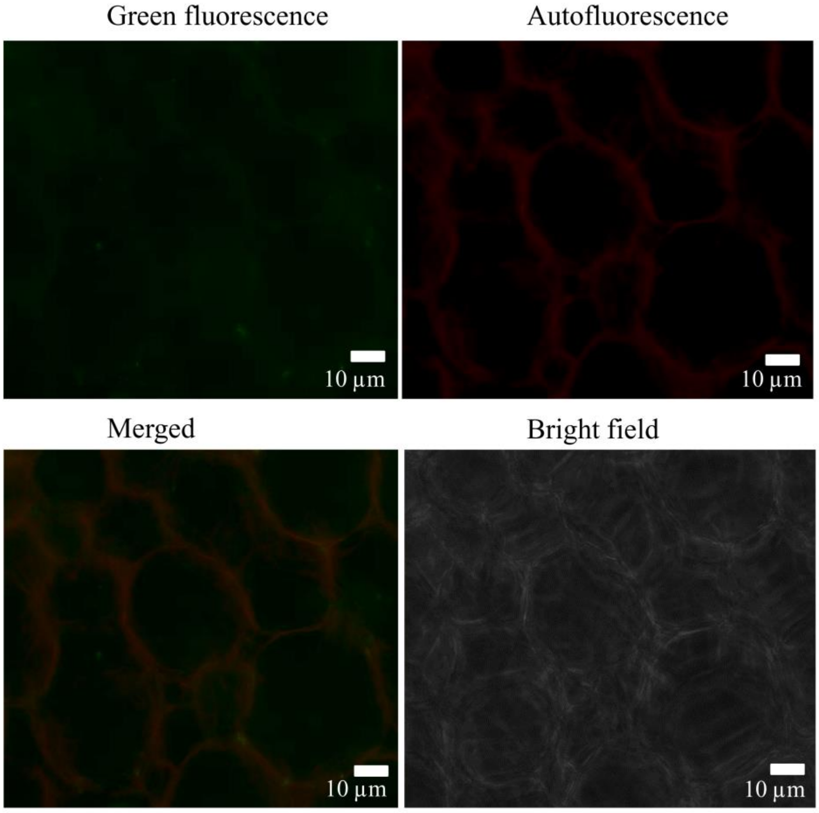

Supplementary Figure S12. Detection of immunological cross-reactivity of anti-OsMMP1 antibody in non-transformed control tobacco plant. Anti-OsMMP1 primary antibody and FITC-tagged secondary antibody were used in this experiment. Stem cross-sections were observed under the epifluorescence microscope (Olympus 1X51, blue excitation, barrier filter 520 nm) and then images were recorded. No significant signal was observed, indicating anti-OsMMP1 antibody is not cross-reactive with endogenous MMPs of tobacco plant. Scale bar = 10 µm.

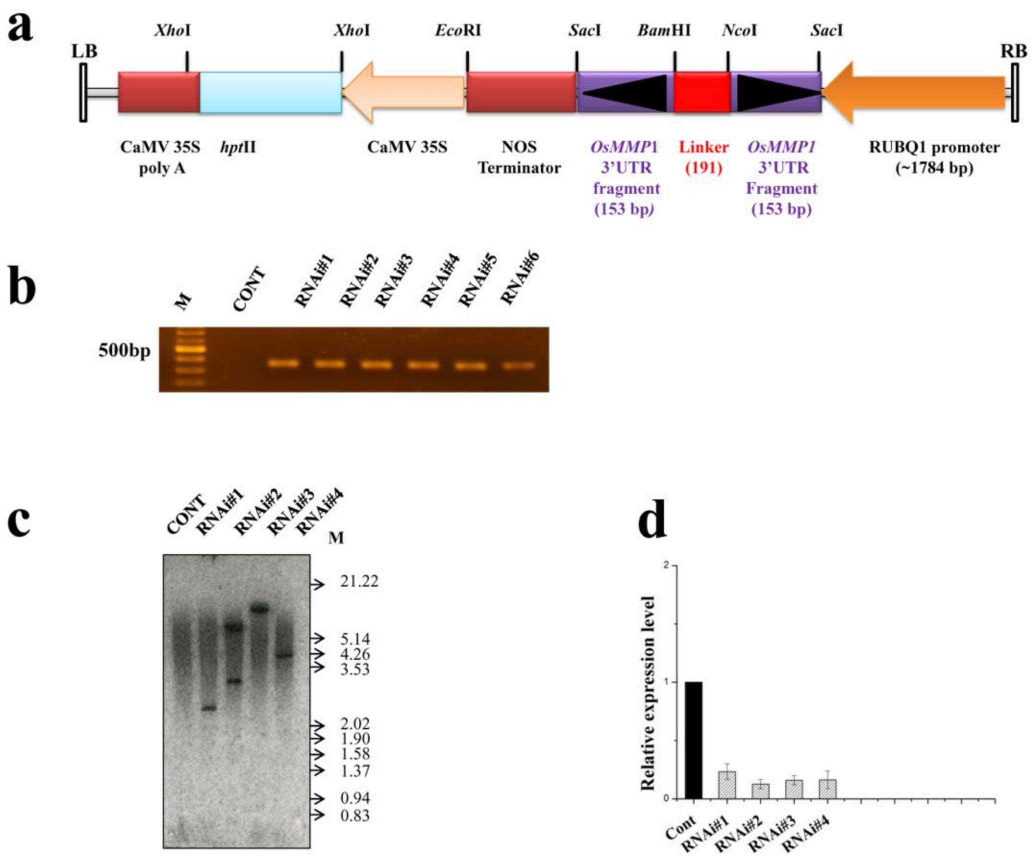

Supplementary Figure S13. Development of independent transgenic rice lines with RNAi-mediated *OsMMP1* gene silencing construct. (a) Schematic diagram of the T-DNA part of pCAM::Rubq/*hp-MMP1*/NOS construct prepared in the pCAMBIA1300 plasmid. **(b)** Ethidium bromide stained 1% agarose gel showing PCR-based screening for the presence of a transgene-specific 364 bp DNA fragment (fusion part of the Rubq promoter and arbitrary linker DNA) in the transgenic rice lines (T3 generation). Lanes M: 100 bp DNA ladder as molecular weight marker, CONT: untransformed control, RNAi#1 to RNAi#6: different transgenic lines. **(c)** Representative Southern blot showing the integration pattern of the transgene in T3 transgenic tobacco lines developed with the pCAM::Rubq/*hp-MMP1*/NOS construct. Lanes CONT: untransformed control, RNAi#1, #2, #3 and #4: four independent T3 transgenic lines, M: arrowheads indicate the position of *Eco*RI and *Hind*III digested lambda DNA as molecular weight marker. (**d**) Relative expression level of *OsMMP1* gene in down-regulated rice lines as determined by qRT-PCR of leaf tissue. Gene expression data were normalized against the internal control rice actin1 gene. Data are expressed as mean ± SE (n=4).

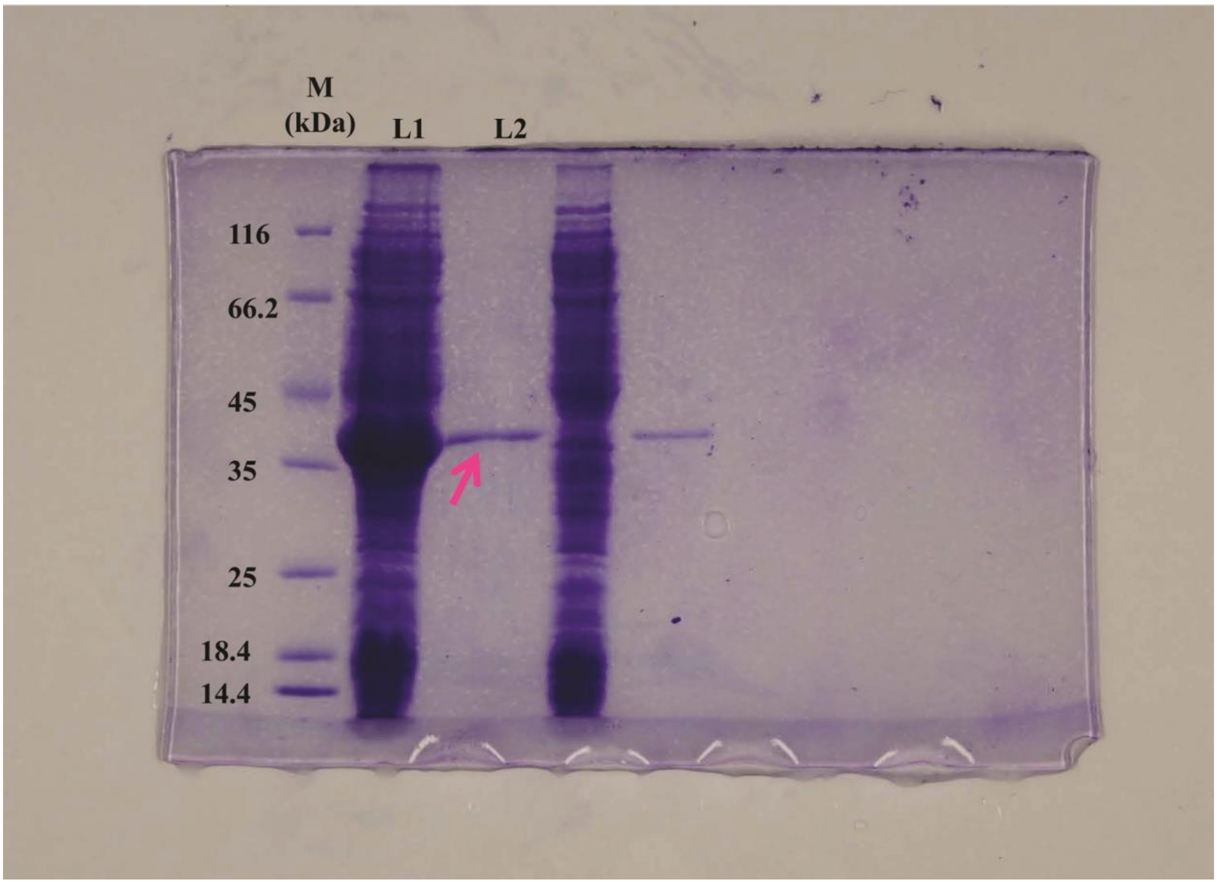

Supplementary Figure S14. Full-length gel of Supplementary Fig. S5I. Purification of the recombinant OsMMP1 (rOsMMP1) protein. Bacterially expressed proteins resolved by 12% SDS-PAGE followed by Coomassie blue staining. Lanes M: protein molecular weight marker, L1: total protein from IPTG induced *E. coli* cells, L2: Ni-NTA purified rOsMMP1 protein (indicated arrow).

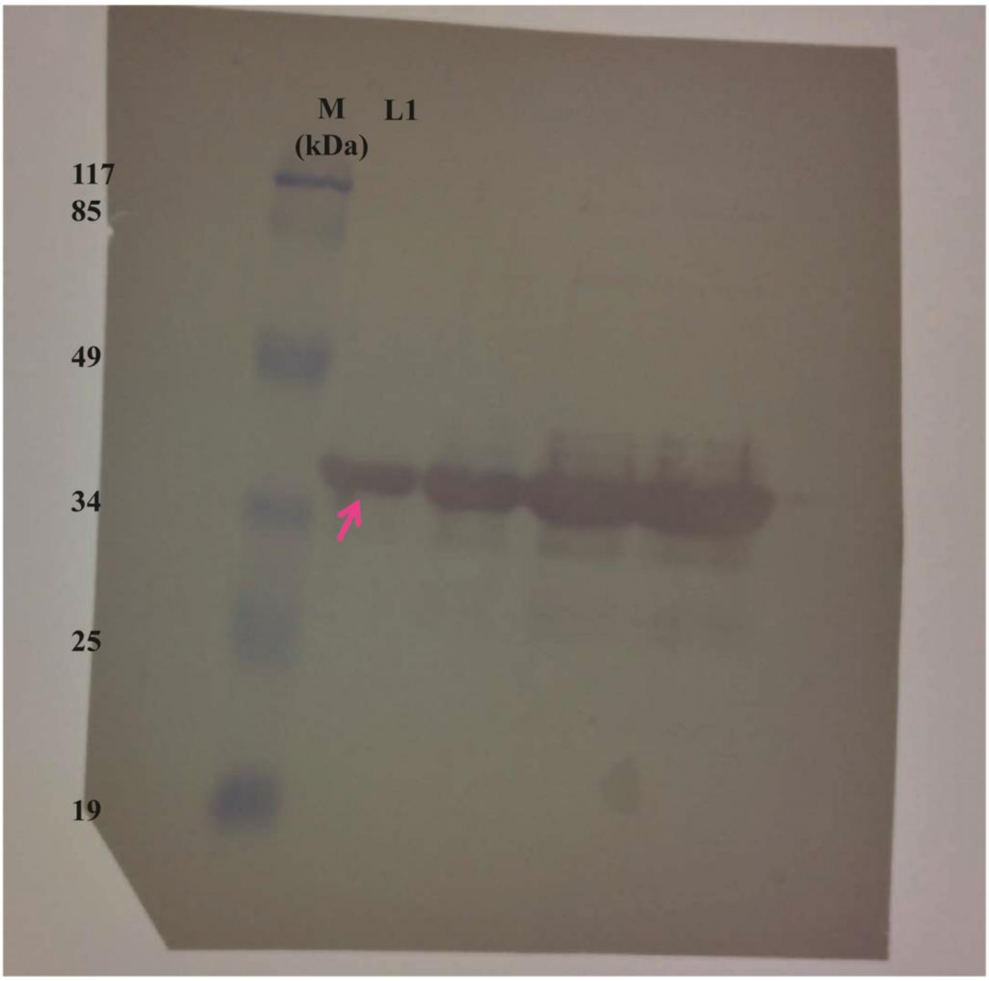

Supplementary Figure S15. Full-length gel of Supplementary Fig. S5III. Western-blot detection of rOsMMP1protein (indicated by arrow) with custom made anti-OsMMP1 antibody.

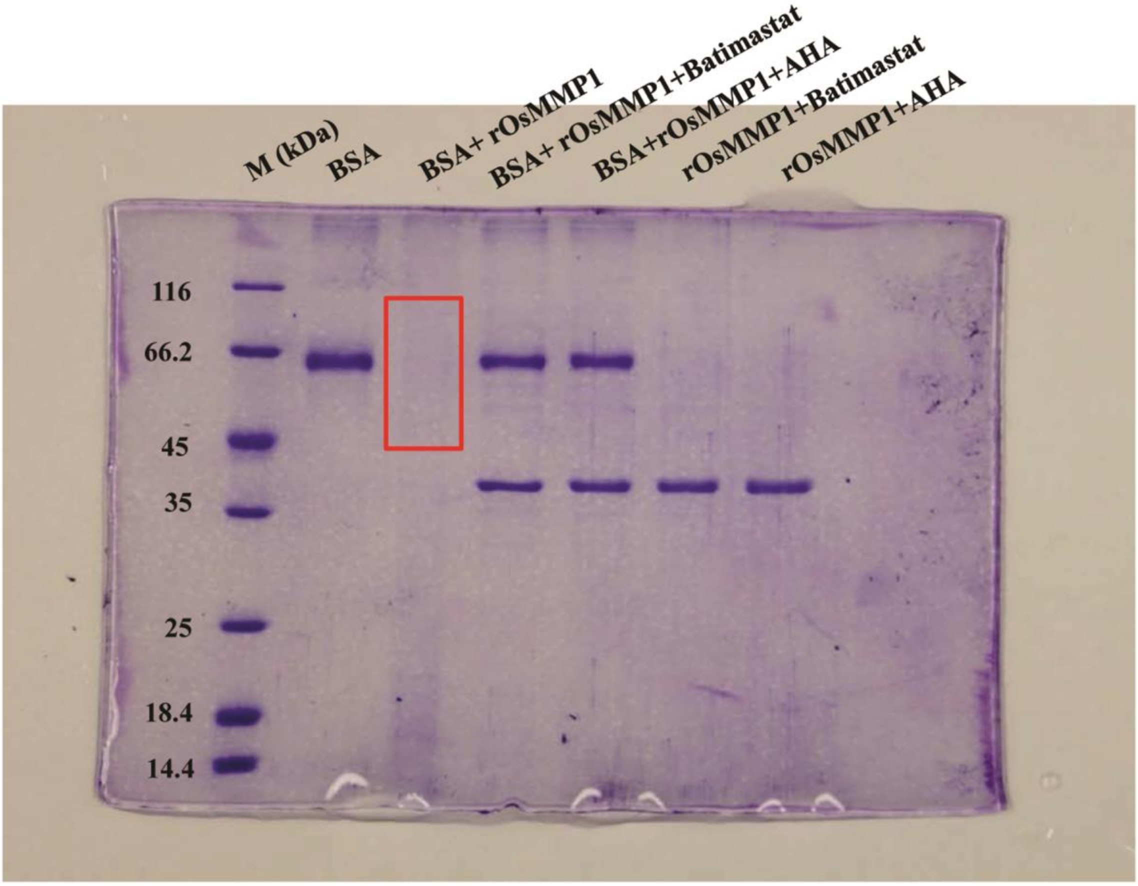

Supplementary Figure S16. Full-length gel of main Fig. 1cI. Analysis of the products formed after protease activity of the rOsMMP1 protein. Rectangular box indicates the proteolytic degradation of BSA.

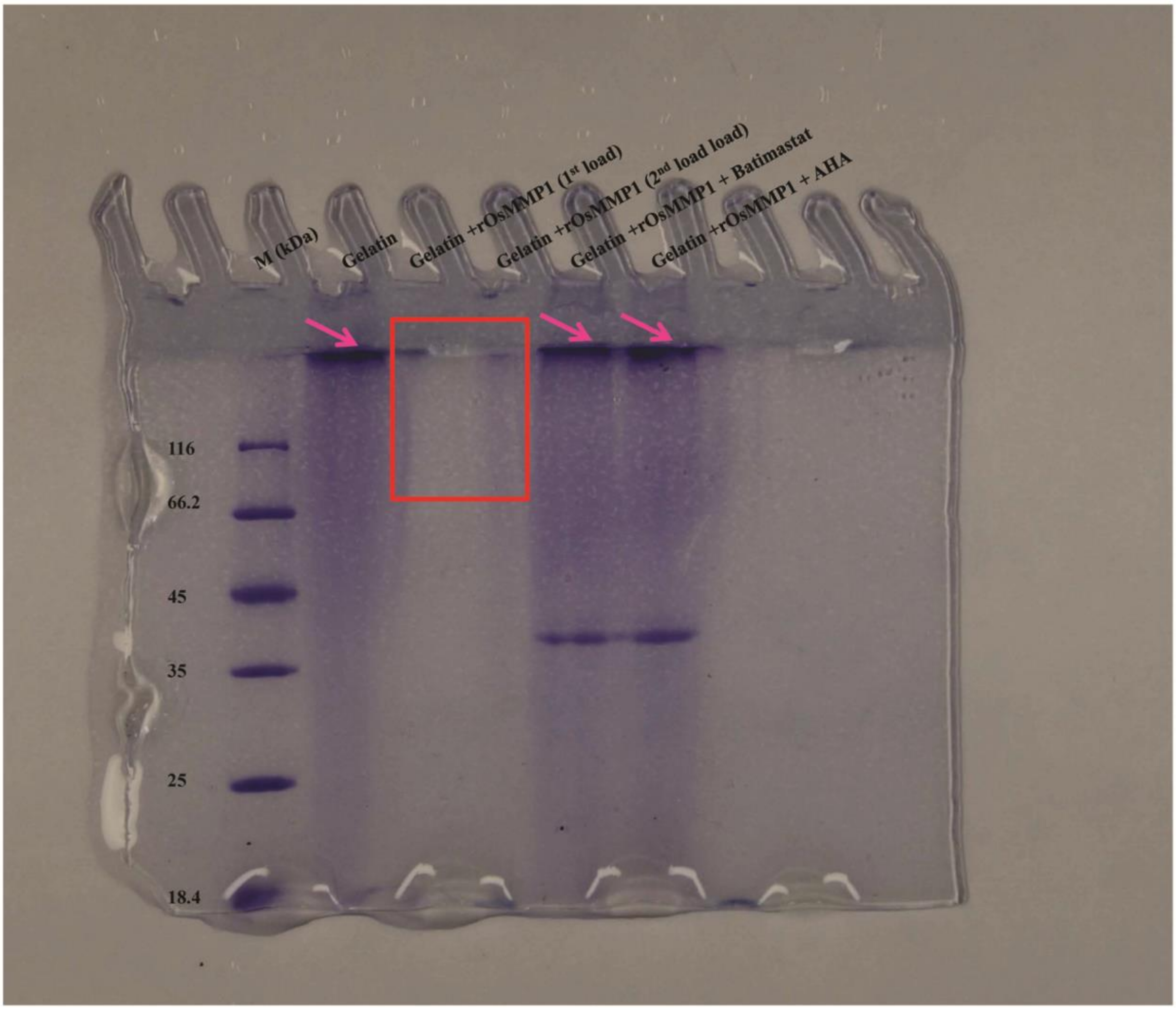

Supplementary Figure S17. Full-length gel of main Fig. 1cII. Analysis of the products formed after protease activity of the rOsMMP1 protein. Rectangular box indicates the proteolytic degradation of gelatin. The arrow indicates the gelatin protein band

Supplementary Table S1. Sequences of primers used in this study

Sl. No.	Primer name	Sequence (5' to 3')	Description
1.	OSMMP1 CDS FORWARD	ATCGGGATCCATGGGCGCCTCCACCTCGCCTCGTC	Used for OsMMP1 CDS cloning and screening of OsMMP1-expressing transgenic tobacco lines.
2.	OSMMP1 CDS REVERSE	GGCCGAGCTCCTACGGAGCTAAGAGCAGG	
3.	OSMMP1 PROMOTER FORWARD	ACGCCTGCAGAGTATCTTCGAACAGGACAC	Used for OsMMP1 promoter cloning
4.	OSMMP1 PROMOTER REVERSE	GCGCGGATCCCATCGCTGAAACCTTGAGCC	
5.	MMP1 /29 FORWARD	ACACGGATCCTTCCCGATGGGATTGCCGGCGA	Used for near-full length OsMMP1 CDS cloning for recombinant rOsMMP1 production
6.	MMP1 /323 REVERSE	GAAAGAGCTCCTACCCGTACAGGCTCTGTA	
7.	MP1 PROM TRANS FORWARD	GCAAGACCACACACACACAT	Screening of *OsMMP1* promoter-*gusA* reporter gene expressing transgenic rice lines.
8.	CATALASE REVERSE	TCATCATCATCATAGACACACGA	
9.	UBQ PROM TRANS FOR	GTCTCATCTTCGCTGGCAAG	Screening of RNAi transgenic rice lines.
10.	FILLER GFP REVERSE	AGAGGGTGAAGGTGATGCAA	
11.	MP1 PROM TRANS FORWARD	GCAAGACCACACACACACAT	Screening of *OsMMP1* promoter-*GFP* reporter gene expressing transgenic tobacco lines.
12.	SMGFP INT REV ERSE	ACGTGTCTTGTAGTTCCCGT	
13.	MP1GSSi3UTR FORWARD	AGACGGATCCATGGCTCCGTAGACTTTCTAGTCCA	Used for cloning of OsMMP1 partial 3'-UTR fragment.
14.	MP1GSSi3UTR REVERSE	GCCGCGAGCTCTATGTATGATATTGATATCTA	
15.	OsMMP1 qPCR FORWARD	TTAGCTCCGTAGACTTTCTAGTCC	Used for qRT-PCR of *OsMMP1*.
16.	OsMMP1 qPCR REVERSE	TGTACACAAGAATCTGTCCAAAAGTC	
17.	RICE ACTIN1 FORWARD	GCAACATCGTTCTCAGTGGTGG	Used for PCR of rice actin1 gene part to detect genomic DNA contamination after DNAseI treatment.
18.	RICE ACTIN1 REVERSE	GCAATCCACATCTGCTGGAATG	
19.	RICE ACTIN1 RT FORWARD	AATTGGATCCGGTATCGTGCTTGACTCTGGT	Used for qRT-PCR of rice actin1 transcript.
20.	RICE ACTIN1 RT REVERSE	GCTCAAGCTTCTGCTGGAAAGTGCTAAGAG	

Supplementary Table S2. Root mean square deviation (RMSD) value of human MMPs used for superimposition with OsMMP1

Sl. No.	Human MMPs	PDB id (from RCSB-PDB database)	RMSD value
1	MMP1	1SU3	0.335 for 157
2	MMP2	1EAK	0.438 for 164
3	MMP3	1G49	0.379 for 175
4	MMP9	1L6J	0.408 for 172
5	MMP10	1Q3A	0.482 for 159
6	MMP13	4G0D	0.330 for 168

Supplementary materials and methods

1. Expression of recombinant OsMMP1 protein in *Escherichia coli*, purification and protease assay

The near full-length CDS of *OsMMP1* covering 29th to 323rd amino acid (without the N-terminal signal peptide and C-terminal transmembrane domain) was PCR amplified and subcloned into an *E. coli* expression plasmid. The forward and reverse primers were designed with incorporated restriction sites *Bam*HI and *Sac*I, respectively for cloning in the pET-28a plasmid having the T7 promoter and 6XHis tag sequence at the 5' end of the cloned *OsMMP1* CDS. The resulting recombinant plasmid pET/OsMMP1 was introduced into *E. coli* BL21 C43 (DE3) cells containing the T7 polymerase gene. A few transformed single colonies were grown in Luria-Bertani medium supplemented with 50 µg ml^{-1} kanamycin at 37 ^{o}C at 200 rpm. Protein expression was induced by additing IPTG with a final concentration of 1 mM. After verifying the recombinant OsMMP1 (rOsMMP1) expression in a few *E. coli* clones, one of them was selected for large-scale rOsMMP1 protein production. Bacterial cells were induced with 1 mM IPTG when OD_{600} reached to 0.6, and the culture continued at 37 ^{o}C for next 4 h. Expressed insoluble 6XHis-tagged rOsMMP1 protein was purified through a Ni-NTA affinity gravity column (Nucleo-pore Ni-NTA agarose bead, Genetix Biotech Asia Pvt. Ltd.) under denaturing condition, following the manufacturer’s instructions. The polyclonal antibodies against His-tagged rOsMMP1 were custom-made in rabbits at IMGENEX India Pvt. Ltd.

For refolding of insoluble rOsMMP1 protein, IPTG-induced bacterial cells from 1 litre culture were harvested by centrifugation (9,000g) at 4 °C and, resuspended in buffer A (20% sucrose, 20 mM Tris-HCl, pH 8, 100 mM NaCl, 1 mM EDTA, 10 mM DTT). The suspension was lysed by ultrasonication keeping on ice, and the lysate was centrifuged at 22,000g for 40 min. The pellet was washed six times with buffer B (10 mM TrisHCl pH 8, 100 mM NaCl, 0.5% Triton-X, 1 mM EDTA, 10 mM DTT), and further washed with buffer C (10 mM TrisHCl pH 8, 100 mM NaCl, 1 mM EDTA, 10 mM DTT). Subsequent washing step was followed (three times) with buffer C without EDTA. Finally, the washed inclusion body was suspended in buffer D (6 M guanidine hydrochloride, 10 mM Na-acetate pH 4.5, and 1 mM DTT) and centrifuged at 22,000g for 15 min. After resuspending in buffer D, the denatured protein (5 mg/ ml) was refolded by rapid dilution (1: 200) method in buffer E (400 mM Arg,

100 mM Tris-HCl pH 8.0, 0.5 mM Glutathione-oxidised, 5 mM Glutathione-reduced). The protein sample was concentrated up to 25 ml and dialyzed extensively against buffer F (50 mM Tris pH 7.5, 20 mM NaCl, 10 mM $CaCl_2$, 1µM $ZnCl_2$). The refolded protein was subjected to size-exclusion chromatography using Superdex 200 prep-grade matrix in a Hiload 16/60 C column (GE Healthcare Biosciences) with buffer F.

Protease activity of the rOsMMP1 was demonstrated by degradation of BSA and gelatin as substrates. The BSA protein is used as a substrate for determining the proteolyic activity of any enzyme[3]. Similarly, the gelatin protein is widely used to assay the proteolytic activity of different MMPs[4]. Gelatin is also a natural substrate of gelatinase, a MMP in animal systems. The Cs1-MMP (from *Cucumis sativus*) has been reported to possess gelatinolytic activity[5]. As BSA and gelatin are widely used for determination of proteolytic activity of MMPs and other proteases, we selected these two proteins for OsMMP1 activity assay. An aliquot of 50 µg of the purified and refolded rOsMMP1 was incubated with 5 µg BSA or 20 µg gelatin in a 40 µl reaction volume for 20 h at 37 °C in digestion buffer (50 mM Tris pH 7.5, 200 mM NaCl, 10 mM $CaCl_2$, 1µM $ZnCl_2$), and products were analyzed through 12% SDS-PAGE to visualize the proteolytic degradation of BSA and gelatin. Inhibition of protease activity was determined by addition of general MMP inhibitors- Batimastat (Sigma) and acetohydroxamic acid (AHA) (Sigma) at the concentration of 2 mM and 50 mM, respectively in the assays.

2. Preparation of transgene construct for RNAi-mediated silencing of *OsMMP1* gene in rice

For RNAi-mediated silencing of *OsMMP1* gene, a hairpin (hp) RNA forming DNA segment was prepared uisng a 156 bp inverted repeat of the partial 3'-UTR fragment of *OsMMP1* gene flanking a 191 bp arbitrary linker DNA, and the hp-MMP1 DNA segment was placed under the rice polyubiquitin 1 (Rubq 1) gene promoter (AY785814). The Rubq1 promoter was first cloned in the pCAMBIA1300 plasmid (Cambia, Australia) at *Hind*III-*Bam*HI orientation, and kept ready for subsequent use. The 3'-UTR fragment of *OsMMP1* gene was PCR amplified using the forward primer (having *Bam*HI and *Nco*I sites) and the reverse primer (having *Sac*I site) (Supplementary Table S1), and cloned in pUC18 plasmid at *Bam*HI-*Sac*I . Next, two DNA fragments of 3'-UTR were obtained from the recombinant pUC18: one as *Bam*HI-*Sac*I fragment and another one as *Nco*I-*Sac*I fragment. Then a ligation reaction was carried out with four DNA fragments: (i) the *Bam*HI-*Sac*I digested 3'-UTR fragment, (ii) *Nco*I-*Sac*I

digested 3'-UTR fragment, (iii) *Bam*HI-*Nco*I digested *GFP* gene (GenBank, U70496) fragment (191 bp) as an arbitrary linker DNA, and (iv) *Sac*I digested pCAMBIA1300 plasmid harbouring the Rubq1 promoter. Thus, the final recombinant binary plasmid pCAM::Rubq/*hp-MMP1*/NOS was developed (Supplementary Fig 13) to introduce into *E. coli* DH10B cells. After isolating the recombinant plasmid DNA from *E coli*, the orientation of different genetic elements was checked through restriction enzyme digestions followed by agarose gel electrophoresis.

3. Preparation of *OsMMP1* promoter-*GFP* reporter gene construct for tobacco transformation

For analysis of the *OsMMP1* promoter function in heterologous tobacco plant, the CDS of the soluble modified red-shifted green fluorescent protein (*smRS-GFP*) gene (GenBank, U70496) was first cloned in the pCAMBIA1300 plasmid (Cambia, Australia) at *Bam*HI-*Sac*I sites. Then the 2 kb putative promoter region of *OsMMP1* gene was cloned at *Pst*I-*Bam*HI in the same plasmid to generate the recombinant plasmid pCAM::proMMP1/*GFP*/NOS (Supplementary Fig S8), which was introduced into *E. coli* DH10B cells. After isolating the plasmid DNA from *E coli*, the orientation of different genetic elements in the recombinant binary plasmid was checked through restriction enzyme digestions followed by agarose gel electrophoresis.

4. Screening of transformed rice and tobacco plants

Following hardening in the glasshouse, a few selected putative transgenic lines of rice and tobacco for each transgene construct were subjected to PCR screening followed by Southern hybridization to confirm the genomic integration of the respective transgene. For Southern hybridization[6], 10-15 μg of genomic DNA from the leaf tissue of control and transgenic lines was digested with suitable restriction enzyme (i.e., *Pst*I for OsMMP1promoter-*GUS* and OsMMP1promoter-*GFP* transgenic lines, and *Bam*HI for RNAi and OsMMP1-overexpressing transgenic lines), and electrophoresed on 1% agarose gel along with molecular weight marker. For OsMMP1promoter-*GUS* and RNAi transgenic rice lines, [α-dCTP^{32}P] labeled 1094 bp fragment of hygromycin resistance (*hptII*) gene was used as hybridization probe. On the other hand, [α-dCTP^{32}P] labeled 2 kb fragment of *OsMMP1* promoter was used as hybridization probe for OsMMP1promoter-*GFP* transgenic tobacco

lines, and [α-dCTP^{32}P] labeled 1094 bp fragment of *hptII* gene was used as hybridization probe for OsMMP1-expressing transgenic tobacco lines.

5. RNA extraction and quantitative reverse transcription PCR (qRT-PCR)

Total RNA was isolated from root and shoot tissues (100 mg starting material) of *indica* rice cultivar IR64, at different developmental stages using RNeasy Plant Mini Kit (Qiagen), according to manufacturer's protocol. Similarly, total RNA was also extracted from leaf tissue of RNAi rice lines and control plants. The RNA sample was quantified in Qubit™ 3 Fluorometer (Thermofischer Scientific). For qualitative check, 1.2% agarose gel electrophoresis was carried out with an aliquot of each RNA sample. RNA samples were treated with DNase I (AMPD1-1KT; Sigma) to remove any genomic DNA contamination. DNase treated RNA samples were quantified again and subjected to PCR reactions (without reverse transcription) with rice actin1 gene primers (flanking the exon3) designed to amplify the part of actin1 gene from the genomic DNA. Absence of any amplicon in the agarose gel confirmed the absence of any genomic DNA in the DNase treated RNA samples. As a positive control, One-Step RT-PCR (Qiagen) reactions were performed with rice actin1 gene primers (forward primer from exon3 and reverse primer from the exon3-exon4 junction) designed to amplify only from the mRNA or cDNA of actin1. These DNase treated RNA samples (100 ng for each reaction) were used for quantitative reverse transcription PCR (qRT-PCR). Primer sets used in qRT-PCR are listed in Supplementary Table S1.

The qRT-PCR was performed in the StepOnePlus Real-Time PCR System (Life Technologies) uisng *Power* SYBR Green RNA-to-CT 1-Step Kit (Life Technologies) followed by melt curve analysis to verify the specificity of PCR amplification. Fold changes were calculated using the comparative CT method[7], and CT values for individual variants were compared to those of an internal reference (rice actin1 transcript).

6. Immunohistochemistry of plant tissues

Sub-cellular localization of OsMMP1 in heterologously expressed tobacco plant was detected following the reported technique[8,9] with minor modification. Different tissue samples (hypocotyls and stem cross-sections) of tobacco were fixed with the prescribed protocol and subjected to blocking with 3% BSA. After blocking, samples were incubated with purified custom-made anti-OsMMP1 primary antibody (1:500 dilutions, IMGENEX India Pvt. Ltd.)

for 16 h at 4 ^{o}C. Subsequently, samples were washed in 1X PBS buffer six times at 15 min interval. Then the samples were incubated with the FITC conjugated anti-rabbit secondary antibody (1:1000 dilutions, G Bioscience) for overnight at 4 ^{o}C. Next, samples were washed well in 1X PBS buffer six times at 15 min interval and mounted on a slide with ProLong Diamond antifade mountant (Molecular Probes). The samples were observed under the epifluorescence microscope (Olympus 1X51, FITC: blue excitation, barrier filter 520 nm; red autofluorescence: green excitation, barrier filter 590 nm), and the images were recorded.

7. Nile Red staining for cuticle

Cuticle layer was detected with reported Nile Red (Sigma) staining method[10]. Stem cross-sections of tobacco were incubated with freshly prepared Nile Red solution (working concentration 1 µg/ml) for 15 min followed by thorough washing in 1X PBS buffer. The prepared sections were observed using epifluorescence microscope (Olympus 1X51, blue excitation, barrier filter 520 nm).

8. Callose detection through aniline blue staining

Qualitative analysis of callose was performed through aniline blue staining protocol reported previously[11] with minor modification. Tobacco leaves and anthers were fixed and bleached with absolute ethanol. The bleached samples were rehydrated through diluted ethanol series (75%, 50%, 25% and 0%). Then the samples were subjected to overnight incubation with 1 M NaOH for tissue softening. Subsequently, samples were treated with 150 mM K_2HPO_4 for 2 h with changes of buffer at 30 min interval. After that, samples were incubated with 150 mM K_2HPO_4 containing 0.03% aniline blue (SRL) for overnight. Leaf sample was simply placed onto a slide, whereas the anther sample was pressed to release pollens. The prepared samples were observed under an epifluorescence microscope (Olympus 1X51, ultraviolet excitation, barrier filter 420 nm). Callose accumulation in pollen was expressed through 3D surface plot showing the fluorescence intensity level of individual pollen using Image-Pro DISCOVERY software.

9. Callose estimation through fluorimetry

The reported protocol of callose quantification through fluorimetry[12] was followed with minor modification. Tobacco tissue was ground to powder with liquid nitrogen. The ground tissue was repeatedly washed with absolute alcohol followed by centrifugation till the

complete removal of green pigment. Next, 500 µl 1 M NaOH solution was added to 5 mg of air-dried sample, and incubated at 80 °C for 15 min to solubilize the callose. Then the extract was centrifuged for 45 min at 15,000g. The callose concentration in the supernatant was quantified fluorometrically using 0.1% (w/v) aniline blue (SRL) at 393 nm excitation and 484 nm emission wavelengths with a spectrofluorometer (Horiba FluoroMax4 spectrometer). Fluorescence intensity of the aniline blue without sample was subtracted from the fluorescence intensity of aniline blue-treated sample to obtain the actual fluorescence of the sample.

References

1 Robert, X. & Gouet, P. Deciphering key features in protein structures with the new ENDscript server. *Nucleic Acids Research* **42**, W320-W324, doi:10.1093/nar/gku316 (2014).

2 Lescot, M. *et al.* PlantCARE, a database of plant cis-acting regulatory elements and a portal to tools for in silico analysis of promoter sequences. *Nucleic Acids Res* **30**, 325-327 (2002).

3 Voss, E. W., Jr., Workman, C. J. & Mummert, M. E. Detection of protease activity using a fluorescence-enhancement globular substrate. *BioTechniques* **20**, 286-291 (1996).

4 Vandooren, J. *et al.* Gelatin degradation assay reveals MMP-9 inhibitors and function of O-glycosylated domain. *World Journal of Biological Chemistry* **2**, 14-24, doi:10.4331/wjbc.v2.i1.14 (2011).

5 Delorme, V. G., McCabe, P. F., Kim, D. J. & Leaver, C. J. A matrix 615 metalloproteinase gene is expressed at the boundary of senescence and programmed 616 cell death in cucumber. *Plant physiology* **123**, 917-927 (2000).

6 Bhattacharya, S., Chattopadhyaya, B., Koduru, L., Das, N. & Maiti, M. K. Heterologous expression of Brassica juncea microsomal ω-3 desaturase gene (BjFad3) improves the nutritionally desirable ω-6:ω-3 fatty acid ratio in rice bran oil.

Plant Cell, Tissue and Organ Culture (PCTOC) **119**, 117-129, doi:10.1007/s11240-014-0518-0 (2014).

7 Livak, K. J. & Schmittgen, T. D. Analysis of relative gene expression data using real-time quantitative PCR and the 2(-Delta Delta C(T)) Method. *Methods* **25**, 402-408, doi:10.1006/meth.2001.1262 (2001).

8 Sauer, M., Paciorek, T., Benkova, E. & Friml, J. Immunocytochemical techniques for whole-mount in situ protein localization in plants. *Nature protocols* **1**, 98-103, doi:10.1038/nprot.2006.15 (2006).

9 Paciorek, T., Sauer, M., Balla, J., Wisniewska, J. & Friml, J. Immunocytochemical technique for protein localization in sections of plant tissues. *Nature protocols* **1**, 104-107, doi:10.1038/nprot.2006.16 (2006).

10 Dobrowolska, I., Majchrzak, O., Baldwin, T. C. & Kurczynska, E. U. Differences in protodermal cell wall structure in zygotic and somatic embryos of Daucus carota (L.) cultured on solid and in liquid media. *Protoplasma* **249**, 117-129, doi:10.1007/s00709-011-0268-3 (2012).

11 Bougourd, S., Marrison, J. & Haseloff, J. Technical advance: an aniline blue staining procedure for confocal microscopy and 3D imaging of normal and perturbed cellular phenotypes in mature Arabidopsis embryos. *The Plant journal : for cell and molecular biology* **24**, 543-550 (2000).

12 Hirano, Y. & Brunner, I. Quantitative determination of callose in tree roots. *Journal of plant physiology* **163**, 1333-1336, doi:10.1016/j.jplph.2005.10.005 (2006).
